# HIF1A regulates follicular atresia through O-GlcNAcylation-mediated VEZF1/ET-1/FOXO1/BAX signaling in porcine granulosa cells

**DOI:** 10.1186/s40104-025-01263-0

**Published:** 2025-09-20

**Authors:** Aiwen Jiang, Jialong Li, Luyao Wang, Yi Liu, Zhengchang Wu, Haifei Wang, Shenglong Wu, Wenbin Bao

**Affiliations:** 1 Key Laboratory for Animal Genetics, Breeding, Reproduction and Molecular Design, College of Animal Science and Technology, Yangzhou University, Yangzhou, 225009 China; 2 Joint International Research Laboratory of Agriculture & Agri-Product Safety, Yangzhou University, Yangzhou, 225009 China

**Keywords:** Follicular selection, Granulosa cell, HIF1A, O-GlcNAcylation, VEZF1

## Abstract

**Background:**

Hypoxic stimuli induce follicular atresia by regulating granulosa cell (GC) apoptosis. Notably, mature follicles can still develop and ovulate under hypoxic conditions, highlighting the importance of the hypoxic adaptation in ovarian follicular selection. To date, the role and mechanism of hypoxia‐inducible factor 1 subunit alpha (HIF1A)-mediated hypoxic responses in follicular atresia are unclear. This study aimed to investigate whether and how HIF1A regulates follicular atresia via the modulation of O-linked N-acetylglucosamine (O-GlcNAc) protein modification (O-GlcNAcylation).

**Results:**

Our findings revealed that HIF1A was highly expressed in pig ovaries. Compared with that in healthy follicles, its expression was significantly downregulated in atretic follicles. Under hypoxic conditions, pharmacological inhibition or siRNA-mediated knockdown of HIF1A increased porcine GC apoptosis. Mechanistically, HIF1A knockdown Suppressed O-GlcNAc transferase degradation, leading to increased global O-GlcNAcylation. Using 4D label-free quantitative proteomics, we identified 53 O-GlcNAcylated proteins. Importantly, O-GlcNAcylation stabilized vascular endothelial zinc finger 1 (VEZF1), and HIF1A knockdown upregulated VEZF1 protein levels by promoting O-GlcNAcylation. The HIF1A-VEZF1 axis modulates forkhead box O1 (FOXO1) expression by regulating endothelin-1. As a transcription factor, FOXO1 directly binds to the Bcl-2 associated X (*BAX*) promoter, activating its transcription and ultimately inducing porcine GC apoptosis and follicular atresia.

**Conclusion:**

Overall, our study elucidates a novel molecular mechanism by which HIF1A deficiency modulates follicular atresia through O-GlcNAcylation-mediated VEZF1 expression. These results not only clarify the molecular mechanism of ovarian follicular development under hypoxic conditions but also offer potential targets for improving follicular selection efficiency in pig breeding.

**Supplementary Information:**

The online version contains supplementary material available at 10.1186/s40104-025-01263-0.

## Introduction

The follicle is the core functional unit of the reproductive system in female mammals, and normal folliculogenesis determines ovarian function, which serves as the basis for follicle maturation and ovulation. Follicular development progresses through primary, secondary, and preovulatory stages, culminating in the release of mature oocytes [1]. However, more than 99% of follicles undergo atresia and degeneration at each stage, leaving fewer than 1% to eventually develop ovulation [2]. Although follicular atresia normally occurs to regulate follicle selection and eliminate abnormal follicles during follicular development, given its significant depletion of follicular resources, it is considered a limiting factor for female reproduction in the development of animal husbandry [3]. In addition, excessive follicular atresia leads to female ovulatory dysfunction, triggering female diseases such as polycystic ovary syndrome (PCOS) and premature ovarian failure (POF) [4–6]. Therefore, elucidating the molecular mechanisms underlying follicular selection (follicular development or follicular atresia) and thereby avoiding excessive follicular atresia is essential both for improving livestock fertility and avoiding female ovarian disorders.

Granulosa cells (GCs) are the most abundant functional cells in follicles. GCs express follicle stimulating hormone receptor (FSHR) and secrete estrogen to promote follicular development [7], and follicular atresia has been shown to be triggered by GC apoptosis [8]. With follicular development, the expanding spatial separation between GCs and the follicular vasculature progressively restricts oxygen diffusion to GCs, thereby establishing a hypoxic microenvironment within the follicle [9]. Accumulating evidence indicates that the hypoxic microenvironment induces follicular atresia by regulating granulosa cell cycle arrest and death [10, 11]; however, mature follicles successfully develop and ovulate under hypoxic conditions, implying that the hypoxic adaptive responses of GCs may determine the process of follicular selection.

Hypoxia-inducible factor 1 subunit alpha (HIF1A) is the most important factor in regulating hypoxic adaptive responses, and its bidirectional function in cellular survival under hypoxic condition has been well documented. On the one hand, the local expression of HIF1A transactivates vascular endothelial growth factor 1 (VEGF1) to regulate angiogenesis, and it can also promote the expression of glucose transporter 1 and activate the glycolytic pathway to maintain cellular energy under hypoxic conditions [12–15]. On the other hand, prolonged HIF1A activation interferes with cellular bioenergetics and biosynthesis, resulting in an energy deficit, which limits proliferation and activates the unfolded protein response [16]. Although the positive function of HIF1A in oocyte maturation and GC growth has been studied [12, 17], the role of HIF1A-mediated hypoxic adaptive responses in determining follicular development or atresia requires further investigation.

O-linked N-acetylglucosamine modification (O-GlcNAcylation) is a dynamic posttranslational modification in which a single O-linked N-acetylglucosamine (O-GlcNAc) is covalently appended to the serine (S) and threonine (T) residues of proteins [18]. This reversible process is governed by two opposing enzymes: O-GlcNAc transferase (OGT) mediates the addition of O-GlcNAc, while O-GlcNAcase (OGA) catalyzes its removal [19]. By modulating protein‒protein interactions, stability, subcellular localization and enzymatic activity, O-GlcNAcylation plays a pivotal role in orchestrating cellular responses to various stressors, including oxidative stress, hypoxia and DNA damage [20]. To date, it remains unclear whether O-GlcNAcylation mediates the role of HIF1A under hypoxic conditions.

In this study, we aimed to explore 1) the role of HIF1A-mediated hypoxic adaptive responses in follicular selection; 2) whether HIF1A regulates O-GlcNAcylation in porcine GCs; and 3) the molecular mechanism by which HIF1A regulates GC apoptosis and follicular atresia via O-GlcNAcylation. Our results demonstrate that HIF1A serves as a critical regulator of follicle selection and protein O-GlcNAcylation. Mechanistically, knockdown of HIF1A disrupts O-GlcNAcylation profiles under hypoxic conditions, leading to significant upregulation of vascular endothelial zinc finger 1 (VEZF1); moreover, the HIF1A-VEZF1 axis mediates GC apoptosis and follicular atresia through the endothelin-1 (ET-1)-forkhead box O1 (FOXO1)-Bcl-2 associated X (BAX) signaling pathway.

## Materials and methods

### Reagents and antibodies

The HIF1A inhibitor PX-478 (HY-10231), the OGT inhibitor OSMI-1 (HY-119738), the OGA inhibitor PugNAc (HY-108241), cycloheximide (CHX, HY-12320) and ET-1 (HY-P0202) were purchased from MedChemExpress (Monmouth Junction, NJ, USA). The antibody products and dilution conditions used in this experiment are shown in Table S1**.**

### Animals

Duroc × Meishan sows (*n* = 3) were used to collect pig tissues, including the heart, liver, spleen, lungs, kidneys, skeletal muscle, fat, ovary and liver. For ovarian follicle and porcine GC isolation, pig ovaries were obtained from Landrace pigs slaughtered at a local slaughterhouse (Xuyi, Anhui, China).

### Follicle isolation and classification

Follicles with a diameter of 3–5 mm were isolated from porcine ovaries via sterile surgical blades. Initially, follicles were roughly divided into healthy follicle (HF) and atretic follicle (AF) groups on the basis of vascular distribution: HF follicles exhibited extensive vascularization, a pinkish appearance, and clear intrafollicular fluid, whereas AF follicles presented no vascularization, a grayish-white appearance, and turbid fluid. Then, the follicular wall was gently peeled off with forceps to release follicular fluid (FF) and internal cells into a 1.5-mL centrifuge tube. A 20-μL aliquot of the mixture was then taken to assess cell viability using trypan blue staining with the Countstar Altair Automatic Cell Analyzer (ALIT Biotech, Shanghai, China). Next, the samples were centrifuged at 1,000 r/min for 5 min at room temperature. The FF was transferred to a new 1.5-mL tube for enzyme-linked immunosorbent assay (ELISA) analysis of 17β-estradiol (E2) and progesterone (P4) levels. Finally, transparent follicles with vascularization, a cell viability > 55% and a P4/E2 ratio < 1 were considered HFs (*n* = 5), whereas opaque follicles with poor vascularization, a cell viability ≤ 55%, and a P4/E2 ratio ≤ 5 were classified as AFs (*n* = 5).

### ELISA assay

The hormone levels of P4 (JL21995, JONLNBIO, Shanghai, China) and E2 (JL10508, JONLNBIO, Shanghai, China) were detected via competitive ELISA. Briefly, in microplates precoated with solid-phase antibodies against porcine E2 or P4, calibrators and FF are added, followed by HRP-labeled P4 or E2 antigen. After incubation and thorough washing to remove unbound components, an immune complex of solid-phase antibody-enzyme-labeled antigen formed on the microplate Surface. Upon the addition of Substrates A and B, the HRP enzyme catalyzes the substrates to generate a blue product, which is converted to yellow by a 2 mol/L Sulfuric acid stop solution. The OD value was measured at 450 nm via a microplate reader (Rayto RT-6100, Shenzhen, China) and was negatively correlated with the concentration of porcine P4 or E2. The sensitivity and accuracy of the ELISAs were reflected by the standard curves (Table S2).

### Immunofluorescence (IF) assay

Fresh porcine ovaries were fixed in 4% paraformaldehyde, embedded in paraffin and then sectioned. After blocking, the sections were incubated with a primary antibody against HIF1A (1:50 dilution) overnight at 4 °C. Next, the sections were incubated for 1 h with secondary antibody. Nuclei were stained with 4',6-diamidino-2-phenylindole dihydrochloride (DAPI) for 15 min. Fluorescence images were captured via a confocal microscope (LSM 710, Zeiss, Oberkochen, Germany).

### Co-staining of HIF1A and TUNEL

Following HIF1A staining, TUNEL staining was performed according to the manufacturer’s instructions (40307ES50, YESEN, Shanghai, China). Briefly, the sample area was completely covered with 100 μL of 1 × equilibration buffer and incubated at room temperature for 30 min. Subsequently, the sections were incubated with TUNEL dye at 37 °C for 60 min. The nuclei were counterstained with DAPI for 15 min, and then, fluorescence images were captured via a Zeiss LSM 710 META microscope.

### Cell culture

Porcine ovaries obtained from a local abattoir (Xuyi, Jiangsu, China) were washed several times with 0.9% sterilized saline supplemented with gentamicin sulfate (1:400, change to: Shanxi Ruicheng Kelong Veterinary Medicine Co., Ltd., China) and 75% ethyl alcohol. Porcine GCs were collected from 3–5 mm antral follicles via aspiration via 10 mL syringes and then inoculated into culture plates filled with DMEM/F12 medium (Gibco, Carlsbad, CA, USA) Supplemented with 15% FBS (Sigma–Aldrich, St. Louis, MO, USA) and 1% penicillin‒streptomycin (PS, Gibco). For porcine GCs isolated by primary culture, the seeding density in a 6-cm culture dish was 1 × 10^6^ cells. The HEK293T cell line was purchased from the Stem Cell Bank of the Chinese Academy of Sciences (Shanghai, China) and cultured with DMEM containing 10% FBS and 1% PS. The cells were incubated at 37 °C in an atmosphere containing 5% CO_2_ and 95% air for the normoxic group, whereas the cells in the hypoxic group were maintained in a 1% O_2_, 5% CO_2_, and 94% N_2_, 37 °C incubator.

### RNA extraction and quantitative real-time PCR (qRT-PCR)

Total RNA was extracted from cultured cells and pig tissues via TRIzol reagent (Invitrogen, Carlsbad, CA, USA). The mRNAs were reverse-transcribed into complementary DNA via PrimeScript RT Master Mix (TaKaRa, Dalian, China) according to the manufacturer’s instructions. qRT‒PCR was performed on a Step-One Plus Real-Time PCR System using AceQ qPCR SYBR Green Master Mix (TakaRa). Relative mRNA levels were calculated via the 2^−△△Ct^ method and normalized against those of *GAPDH*. All primers were synthesized by GENEWIZ (GENEWIZ, Suzhou, China). The primer sequences are listed in Table S3.

### Plasmids, RNA oligonucleotides and cell transfection

A 2000-bp wild-type (WT) *BAX* promoter sequence was amplified and inserted into a pGL3-basic reporter (Promega, Madison, WI, USA) between the Nhe I and Hind III enzyme sites to generate a luciferase reporter (pGL3-*BAX*-WT). We subsequently mutated the FOXO recognition elements (FREs) upstream of the luciferase reporter gene, generating the mutation (MUT) constructs pGL3-*BAX*-M1, pGL3-*BAX*-M2, and pGL3-*BAX*-M3 via a mutagenesis kit (Vazyme, Nanjing, China). The primers used to construct the luciferase reporters are shown in Table S4. The Flag-*VEZF1*-WT plasmid, Flag-*VEZF1*-MUT plasmid, *HIF1A* siRNAs, *OGT* siRNAs, *VEZF1* siRNAs, *FOXO1* siRNAs and *BAX* siRNAs were purchased from GenePharma (Shanghai, China). Vectors for the *HIF1A*, *FOXO1*, *OGT* and *BAX* overexpression plasmids, as well as the pGL3-*EDN1*-WT and pGL3-*EDN1*-MUT constructs, were synthesized by Tsingke (Nanjing, China). Transfection was performed via the Lipofectamine 3000 reagent (Invitrogen, Carlsbad, CA, USA) according to the manufacturer’s instructions.

### Apoptosis analysis

After primary porcine GCs adhered to the culture plate, they were Subjected to hypoxic treatment, the addition of 50 µmol/L PX478 or 100 nmol/L ET-1, or transfection with *HIF1A* siRNA, *VEZF1* siRNA, *BAX* siRNA, the *FOXO1* overexpression plasmid, or the *BAX* overexpression plasmid. At 24 h after treatment, porcine GCs were trypsinized and washed twice with PBS. Then, the cells were resuspended in 500 μL of binding buffer and incubated with 5 µL of Annexin V-FITC and 5 µL of propidium iodide (PI) for 5 min in the dark. The samples were assessed on a CytoFLEX (Beckman Coulter, Inc., CA, USA) or FACSCalibur flow cytometer (Becton Dickinson, San Diego, CA, USA). The data were analyzed via CytExpert or FlowJo software. A total of 10,000 cells were analyzed per sample.

### Protein extraction and western blot analysis

Total protein was extracted via RIPA buffer (Applygen, Beijing, China) Supplemented with 1 mmol/L phenylmethylsulfonyl fluoride (PMSF; Beyotime, Shanghai, China). The protein concentration was determined via a BCA protein assay kit (Beyotime, Shanghai, China). Western blotting was performed to investigate protein expression. Protein denaturation was performed by boiling the cell lysates for 10 min in SDS loading buffer (Biosharp, Shanghai, China). Briefly, samples (each containing 30 μg of protein) were electrophoresed on a 4%–20% ExpressPlus PAGE gel (GeneScript, Nanjing, Jiangsu, China) and transferred to a PVDF membrane (Millipore, Sigma). The membrane was Subsequently blocked with 5% bovine serum albumin (Millipore, Sigma) prepared in Tris-buffered saline containing Tween-20 for 2 h at room temperature. Thereafter, the membrane was incubated overnight at 4 °C with diluted antibodies. Next, the membrane was incubated with HRP–conjugated goat anti-rabbit or anti-mouse IgG antibodies (Cowin Biosciences, Jiangsu, China) for 2 h at room temperature. The signals were visualized via WesternBright ECL Chemiluminescent HRP substrate (Advansta, Bering Drive, San Jose, CA, USA) and an ImageQuant LAS-4000 system (Fujifilm, Tokyo, Japan). Signal intensities were analyzed via ImageJ software. Relative protein level is reflected by the ratio of the gray intensities of the target proteins to that of the internal reference protein (GAPDH).

### Co-immunoprecipitation (Co-IP) analysis

Porcine GCs washed with PBS were lysed on ice with IP lysis buffer (Pierce, 26149) containing a protease inhibitor cocktail (Roche, 04693132001). The lysate was centrifuged at 14,000 × *g* for 15 min at 4 °C, and the supernatant was collected. Whole-cell lysates (WCLs) were then Subjected to IP. The target protein-specific antibodies were added to 500 μL of WCL according to Table S1, followed by overnight incubation with rotation at 4 °C. After adding 25 μL of protein A/G magnetic beads (88802, Thermo Fisher Scientific), the mixture was incubated for an additional 1 h at 4 °C. The beads were pelleted via a magnetic separator, and the Supernatant was discarded. The immunoprecipitates were washed with 1× cell lysis buffer, magnetized again to remove the supernatant, and eluted with SDS loading buffer (SunShineBio, Nanjing, China), followed by immunoblotting (IB) analysis. The antibody correspondence table for IP and IB is shown in Table S5.

### O-GlcNAcylation 4D-label-free quantitative proteomic

Porcine GCs were seeded into 15-cm sterile culture dishes under 20% O_2_. After cell attachment, the cells were cultured under 1% O_2_ for an additional 24 h. Following the 24 h incubation, the cells were washed three times with PBS. Subsequently, 2 mL of PBS was added to each 15-cm dish, and the cells were gently scraped off via a cell scraper and collected into a 15-mL centrifuge tube. The cell Suspension was centrifuged at 1,000 × *g* for 5 min at room temperature, and the supernatant was discarded. The cell pellet was then sent to Shanghai Applied Protein Technology Company (Shanghai, China) for analysis. Briefly, total protein extracted from the tissues was digested into peptides via trypsin. The peptides were subsequently incubated with anti-GlcNAc S/T antibody beads (PTMScan O-GlcNAc [GlcNAc-S/T] Motif Kit; Cell Signaling Technology) with gentle shaking overnight. The beads were washed three times with cold IPA buffer and cold water, followed by desalting via ZipTip. Finally, the samples were separated for LC‒MS/MS detection.

### Chromatin immunoprecipitation (ChIP) and ChIP-qPCR analysis

All ChIP samples were collected from 10-cm cell culture dishes. Briefly, the cell pellet was isolated after treatment with 1% formaldehyde and 0.125 mol/L glycine solution; then, micrococcal nuclease was used to digest the chromatin at a concentration of 0.06 U/μL. For immunoprecipitation, digested chromatin was mixed with FOXO1 antibodies separately at 4 °C overnight. Finally, the purified DNA was collected after IP elution and DNA recovery, and PCR or qRT-PCR was conducted to detect changes in the *BAX* promoter. The *BAX* primer sequences for ChIP or ChIP-qPCR were as follows: F: 5'-TTGATACAGCGTAGCAGC-3'; R: 5'-ATTTGAAGGAAGAGTGGG-3'.

### Preparation of nuclear and cytosolic protein lysates

Nuclear‒cytoplasmic fractionation of porcine GCs was performed to isolate nuclear and cytosolic proteins, followed by western blot analysis of FOXO1 expression. Cytosolic and nuclear extracts were prepared according to the manufacturer’s instructions for the NE-PER Nuclear and Cytoplasmic Extraction Reagents (Thermo Fisher Scientific, 78833). Briefly, GCs were washed twice with ice-cold PBS buffer and centrifuged at 500 × *g* for 3 min. After the addition of Cytoplasmic Extraction Reagent I, the lysate was incubated on ice for 10 min, and Cytoplasmic Extraction Reagent II was added to the Suspension, which was then centrifuged at 16,000 × *g* at 4 °C for 5 min. The supernatant was collected as the cytoplasmic extract. The insoluble (pellet) fraction was resuspended in Nuclear Extraction Reagent, incubated on ice for 40 min, and then centrifuged at 16,000 × *g* at 4 °C for 10 min. The supernatant was collected as the nuclear extract. Both nuclear and cytosolic extracts were used for subsequent western blotting experiments.

### Dual luciferase assay

HEK293T cells were seeded into 6-well plates, and then, the PGL3-*BAX* luciferase reporters were cotransfected with pRL-TK and pcDNA3.1-FLAG-*FOXO1* or the control pcDNA3.1 into HEK293T cells. At 48 h after transfection, the firefly and Renilla activities were determined on a Glomax^®^ 20/20 luminometer (Promega, Madison, WI, USA) via the Dual-Luciferase® Reporter Assay System (Promega). The ratio of Renilla luciferase activity to firefly luciferase activity was calculated.

### Protein degradation analysis

After the cells were transfected with *HIF1A* siRNAs or treated with 50 μmol/L OSMI-1 and 10 μmol/L PugNAc for 12 h, 100 nmol/L CHX was added to inhibit the protein synthesis pathway. After CHX was added, protein samples were collected at different time points. The protein samples from both the treatment and control groups without CHX addition were normalized to 100%, and the differences in remaining protein levels between the treatment and control groups at the same time points were analyzed.

### Statistical analysis

Statistical analyses were performed via Prism 6 software (GraphPad Software, La Jolla, CA, USA). The results are expressed as the mean ± standard deviation (SD). The number of biological replicates is indicated by dots in the statistical graphs. Comparisons between two groups were analyzed using the two-tailed unpaired Student’s *t*-test. For data involving three or more groups, one-way analysis of variance (ANOVA) combined with Tukey’s post-hoc test was applied. *P* < 0.05 was considered significant. Pearson’s correlation analysis was performed to evaluate the relationships between *HIF1A* and follicle development-related genes, including *FSHR* and estrogen receptor 1 (*ESR1*), androgen receptor (*AR*), and cytochrome P450 family 11 Subfamily A member 1 (*CYP11A1*).

## Results

### HIF1A is negatively associated with follicular atresia

**Fig. 1:**
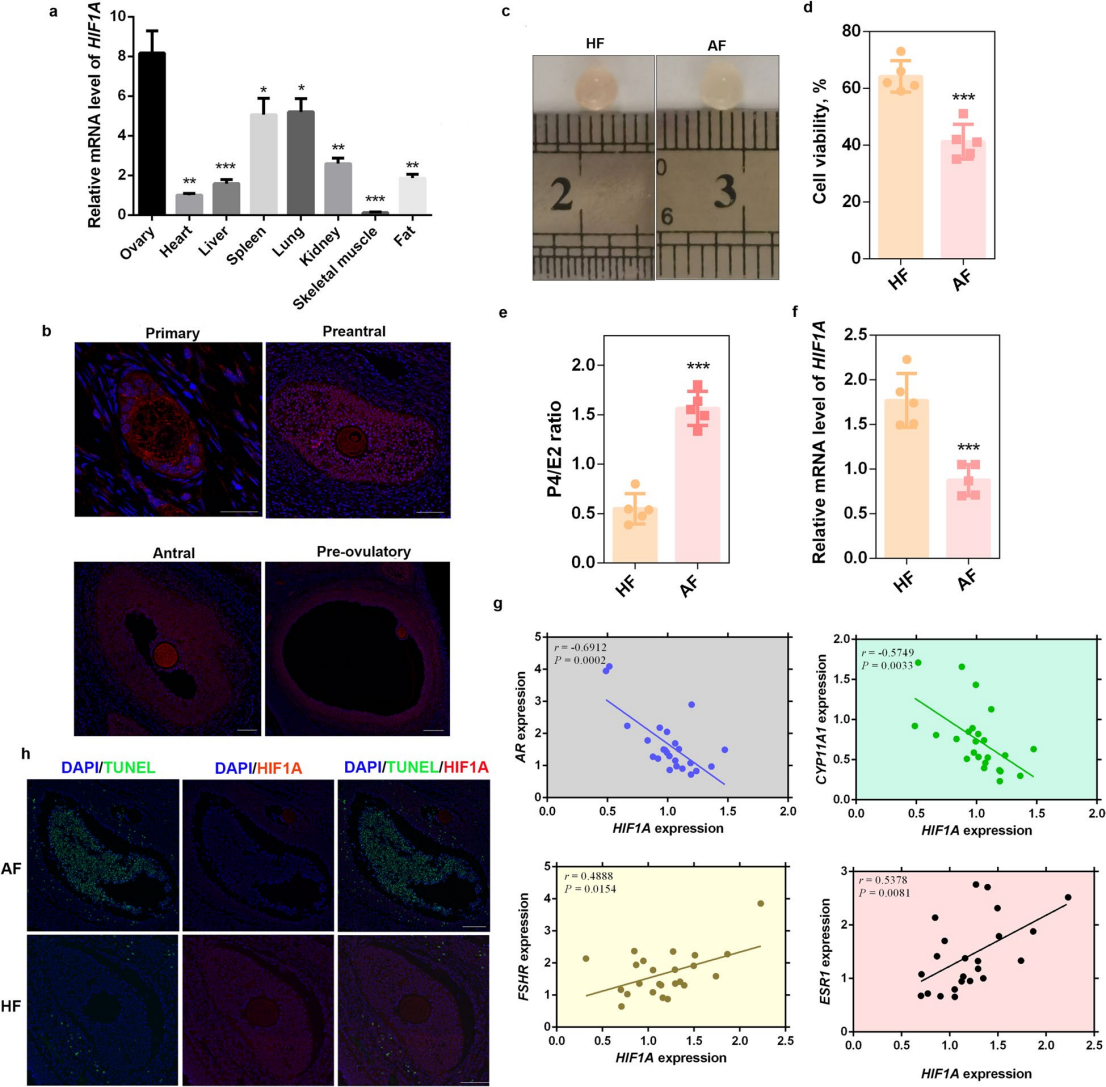
HIF1A is negatively associated with follicular atresia. **a** The mRNA levels of *HIF1A* in multiple porcine tissues were determined. *n* = 3 biological replicates per group.** b** The expression patterns of HIF1A throughout the folliculogenesis process were detected via IF. HIF1A-positive cells were stained red, and nuclei were stained with DAPI (blue). Scale bar, 100 μm.** c** Representative images of follicles in the HF and AF groups.** d** Cell viability analysis between the HF and AF groups. *n* = 5 biological replicates per group.** e** P4/E2 ratios in the HF and AF groups. *n* = 5 biological replicates per group. **f** The differences in the mRNA levels of *HIF1A* between the HF and AF groups were determined. *n* = 5 biological replicates per group. **g** Correlation analysis between *HIF1A* and *AR*, *CYP11A1*, *FSHR*, and *ESR1* mRNA levels in porcine GCs. *n* = 23 biological replicates per group.** h** Costaining analysis of HIF1A and TUNEL in porcine ovaries. HIF1A-positive cells were stained red, TUNEL-positive cells were stained green, and nuclei were stained with DAPI (blue). Scale bar, 100 μm. ^*^P < 0.05, ^**^P < 0.01, ^***^P < 0.001

To explore the association between HIF1A and follicular fate, we first characterized its expression pattern across different porcine tissues. Our results revealed that the expression level of *HIF1A* in the ovary was significantly greater (*P* < 0.05) than that in other tissues (Fig. 1a). In addition, immunofluorescence analysis revealed that HIF1A was consistently expressed throughout the folliculogenesis process and was widely distributed in the GC layer and oocytes (Fig. 1b), suggesting its important role in normal follicular development. Then, HFs and AFs were isolated according to their vascular distribution (Fig. 1c), cell viability (Fig. 1d), and P4/E2 ratio (Fig. 1e). The results revealed that *HIF1A* expression was significantly lower (*P* < 0.001) in the AF group than in the HF group (Fig. 1f). More importantly, we analyzed the correlation between *HIF1A* and key follicle development-related genes, including *FSHR*, *ESR1*, *AR* and *CYP11A1*. The results revealed that *HIF1A* was positively correlated with *FSHR* (*r* = 0.4888, *P* = 0.0154) and *ESR1* (*r* = 0.5378, *P* = 0.0081), which are critical regulators of estrogen synthesis and follicular development, whereas it was negatively correlated with *AR* (*r* = −0.6912, *P* = 0.0002) and *CYP11A1* (*r* = –0.5749, *P* = 0.0033), which are involved in androgen metabolism and progesterone production (Fig. 1g). To further investigate the direct evidence that HIF1A is associated with follicles fate, we performed HIF1A and TUNEL co-staining on porcine ovaries. The results revealed that the TUNEL fluorescence intensity in AFs was significantly greater (*P* < 0.001) than that in HFs, whereas the HIF1A fluorescence intensity was significantly lower (*P* < 0.01) than that in HFs (Fig. 1h and Fig. S1).

### Knockdown of HIF1A promotes porcine granulosa cell apoptosis

**Fig. 2:**
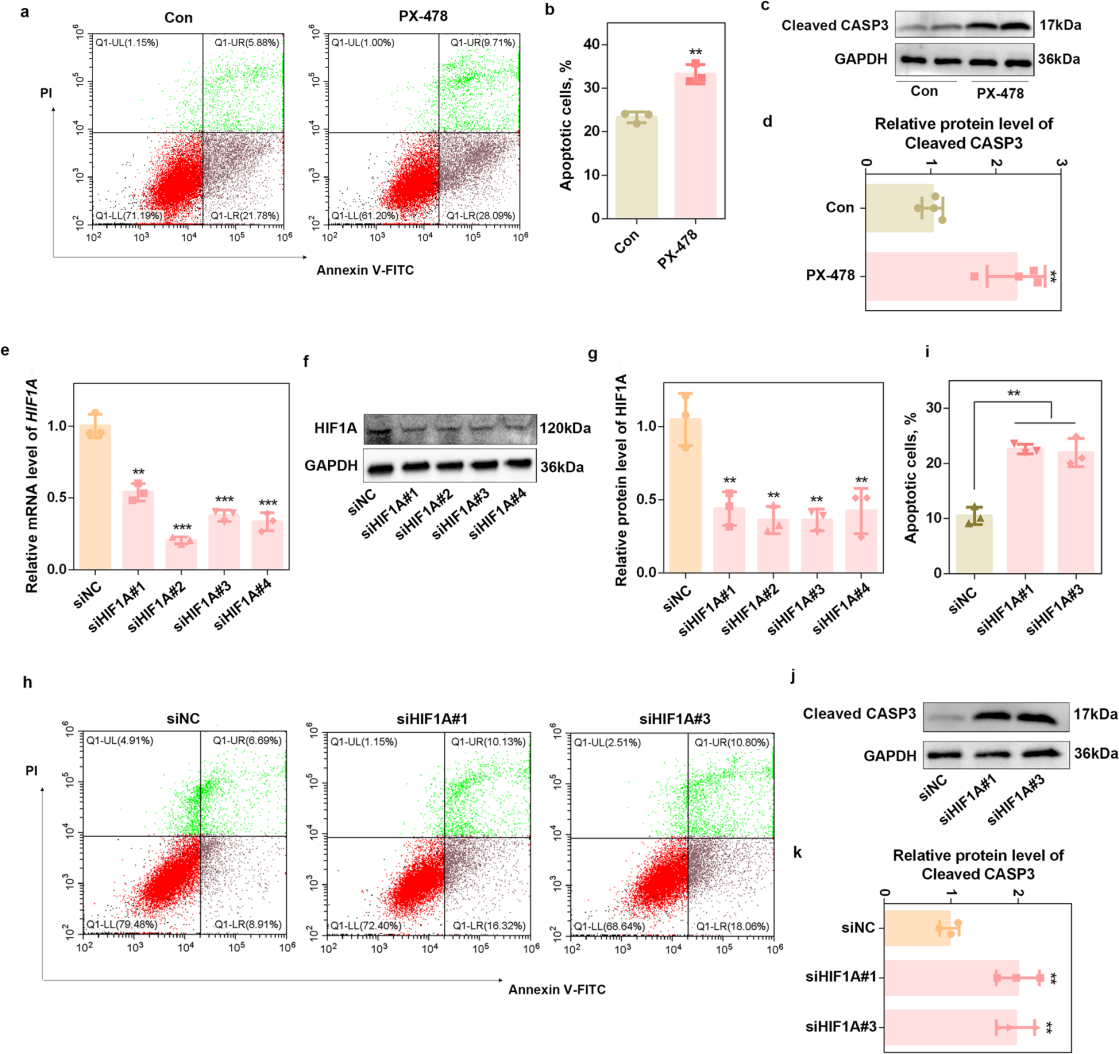
Knockdown of HIF1A promotes porcine granulosa cell apoptosis.** a** and **b** The percentage of apoptotic porcine GCs was determined after PX-478 treatment under hypoxic conditions. *n* = 3 biological replicates per group. **c **and** d** Western blot analysis was performed to detect cleaved CAPS3 expression after porcine GCs were treated with PX-478 under hypoxic conditions. *n* = 3 biological replicates per group.** e** The knockdown efficiency of *HIF1A* mRNA was determined after transfection with HIF1A siRNAs under hypoxic conditions. *n* = 3 biological replicates per group.** f** and **g** Western blot analysis was performed to detect HIF1A expression after porcine GCs were transfected with HIF1A siRNAs under hypoxic conditions. *n* = 3 biological replicates per group. **h** and **i** The apoptotic rate of porcine GCs was determined after siHIF1A#1 and siHIF1A#3 transfection under hypoxic conditions.* n* = 3 biological replicates per group. **j** and **k** Western blot analysis was performed to detect cleaved CAPS3 expression after siHIF1A#1 and siHIF1A#3 transfection under hypoxic conditions.* n* = 3 biological replicates per group. ^**^P < 0.01, ^***^P < 0.001

Granulosa apoptosis was found to be the key determinant of follicular atresia [8]. To investigate the role of HIF1A in follicular atresia and granulosa apoptosis, porcine GCs were isolated and cultured under hypoxic conditions. Our results revealed that hypoxic treatment significantly increased the protein level of HIF1A (*P* < 0.01) and led to a marked increase in the apoptosis of granulosa cells (*P* < 0.01) (Fig. S2). A ‘loss-of-function’ experiment was subsequently performed with PX-478, a specific HIF1A inhibitor, under hypoxic conditions. As shown in Fig. 2a and b, PX-478 significantly increased the proportion of apoptotic GCs (*P* < 0.01). Western blot analysis further confirmed that PX-478 upregulated the expression of cleaved CASP3, an apoptotic marker protein (*P* < 0.01) (Fig. 2c–d). Next, *HIF1A* expression was knocked down via siRNA (siHIF1A) transfection. All four siRNAs significantly reduced the HIF1A mRNA and protein levels (*P* < 0.01) (Fig. 2e–g). Then, siHIF1A#1 and siHIF1A#3 were transfected into porcine GCs to measure apoptosis rates. Consistent with PX-478 treatment, transfection of siHIF1A#1 and siHIF1A#3 increased the percentage of apoptotic porcine GCs (*P* < 0.01) (Fig. 2h–i). Accordingly, the protein level of cleaved CASP3 was significantly increased (*P* < 0.01; Fig. 2j–k). These results suggest that the hypoxic response capacity of GCs, which is primarily mediated by HIF1A, is a critical regulator of GC apoptosis and follicular atresia.

### Knockdown of HIF1A disrupts O-GlcNAcylation by increasing OGT protein levels

**Fig. 3:**
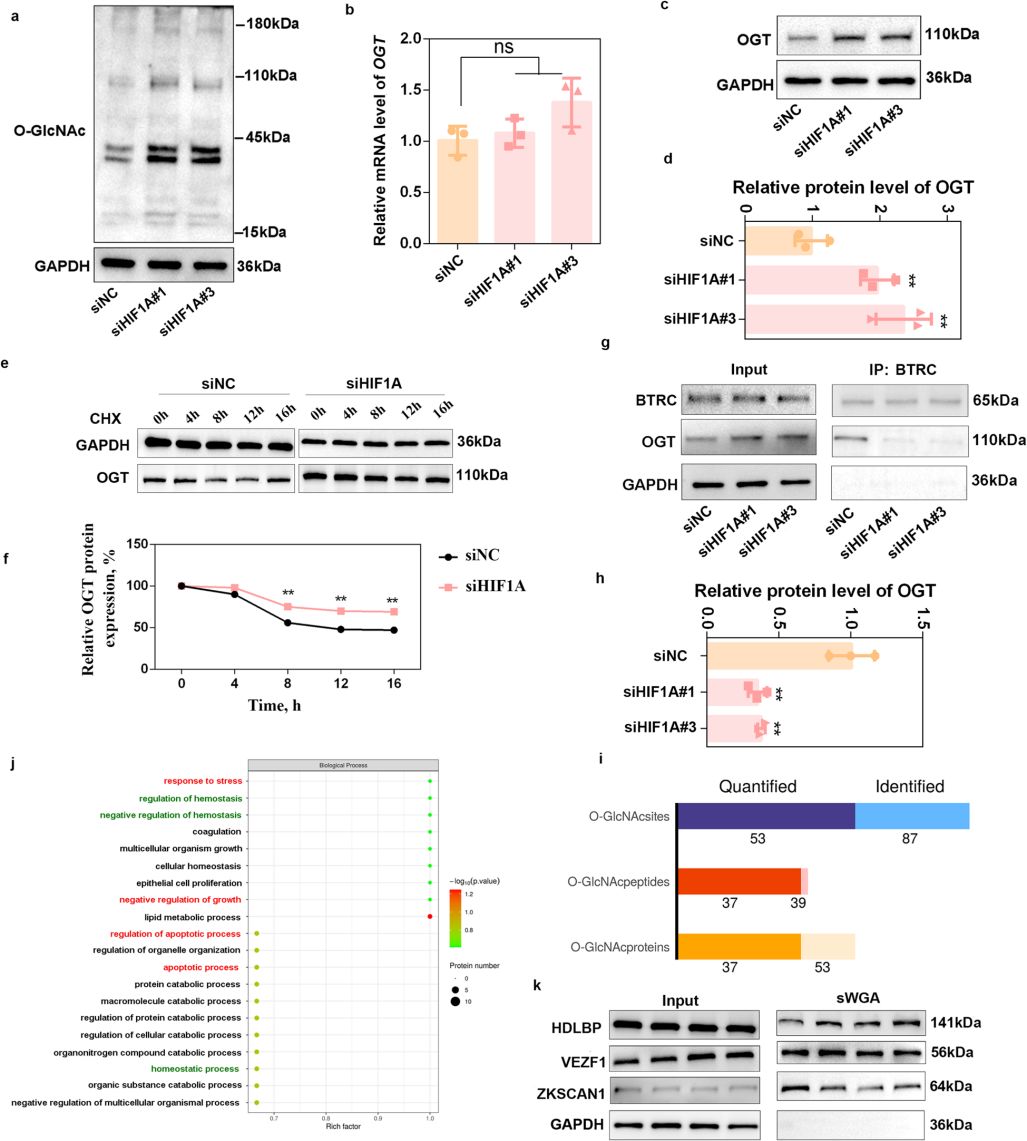
Knockdown of HIF1A disrupts O-GlcNAcylation by increasing OGT protein levels. **a** Western blot analysis was performed to detect O-GlcNAcylation levels after siHIF1A#1 and siHIF1A#3 transfection under hypoxic conditions. *n* = 3 biological replicates per group. **b** The mRNA levels of *OGT* were determined after siHIF1A#1 and siHIF1A#3 transfection under hypoxic conditions. *n* = 3 biological replicates per group. **c** and **d** Western blot analysis was performed to detect OGT expression after siHIF1A#1 and siHIF1A#3 transfection under hypoxic conditions. *n* = 3 biological replicates per group. **e** and **f** Analysis of the OGT protein degradation rate after transfection with siHIF1A. Porcine GCs transfected with siHIF1A were treated with CHX, and the protein level of OGT was recorded at 0, 4, 8, 12 and 16 h by western blotting. **g** and **h** Co-IP analysis was performed to detect the combination of BTRC with OGT after siHIF1A#1 and siHIF1A#3 transfection under hypoxic conditions. *n* = 3 biological replicates per group. **i** Summary of O-GlcNAcylated peptides that were quantified and identified in porcine GCs under hypoxic conditions. **j** Biological process enrichment of the quantified O-GlcNAcylated proteins. **k** Porcine GCs were collected to perform IP with sWGA agarose, which selectively binds to O-GlcNAc to valide O-GlcNAcylated peptides. ^**^P < 0.01; ns, not significant

Studies have shown that O-GlcNAcylation is sensitive to hypoxic stimuli [20, 21]. As shown in Fig. S3a–d, while lysine lactylation of proteins (Pan-Kla) was stably upregulated (*P* < 0.01) after hypoxic treatment, O-GlcNAcylation levels were differentially regulated, with some proteins upregulated and some downregulated (*P* < 0.05). Next, we detected O-GlcNAcylation after HIF1A knockdown. The results revealed that transfection of siHIF1A#1 and siHIF1A#3 led to the dysregulation of O-GlcNAcylation under hypoxic conditions, resulting in a significant increase in the global O-GlcNAcylation level (*P* < 0.01) (Fig. 3a and Fig. S3e). As OGT is the sole enzyme responsible for O-GlcNAcylation in mammalian cells, we hypothesized that the aberrant increase in global O-GlcNAcylation following siHIF1A transfection might be attributed to altered OGT expression. Our results revealed that knockdown of HIF1A did not change the mRNA level of *OGT* (*P* > 0.05) (Fig. 3b); however, both siHIF1A#1 and siHIF1A#3 significantly upregulated OGT protein levels (*P* < 0.01) (Fig. 3c–d), which was consistent with the increase in the global O-GlcNAc level and indicated that HIF1A regulates OGT expression at the posttranslational level. We then treated porcine GCs with CHX to inhibit protein synthesis, and the results revealed that, compared with the siNC treatment, HIF1A knockdown delayed the degradation of OGT (Fig. 3e–f). Liu et al*.* [22] reported that the degradation of OGT specifically relies on the beta-transducin repeat containing protein (BTRC)-mediated proteasomal pathway under hypoxic conditions. Co-IP analysis revealed that HIF1A knockdown significantly decreased the combination of OGT with BTRC (*P* < 0.01) (Fig. 3g–h), further confirming that HIF1A regulates O-GlcNAcylation by BTRC-mediated OGT expression.

To identify the O-GlcNAcylated proteins that are regulated by HIF1A in porcine GCs, we performed 4D-label-free quantitative O-GlcNAcylation following hypoxic exposure. A total of 87 unique O-GlcNAcylation sites across 53 proteins were identified (Fig. 3i and Table S6). Biological process enrichment revealed that these O-GlcNAcylated proteins were involved in “response to stress”, “negative regulation of growth”, “regulation of hemostasis”, “regulation of apoptotic process” and “apoptotic process” (Fig. 3j), indicating their potential role in regulating follicular selection and cellular homeostasis under hypoxic conditions. Moreover, affinity chromatography using succinylated wheat germ agglutinin (sWGA) beads, which selectively bind to O-GlcNAc, validated the accuracy of the sequencing data by detecting the combination of O-GlcNAc with high-density lipoprotein binding protein (HDLBP), zinc finger protein with KRAB, SCAN domains 1 (ZKSCAN1) and VEZF1 (Fig. 3k).

### O-GlcNAcylation stabilizes the VEZF1 protein

**Fig. 4:**
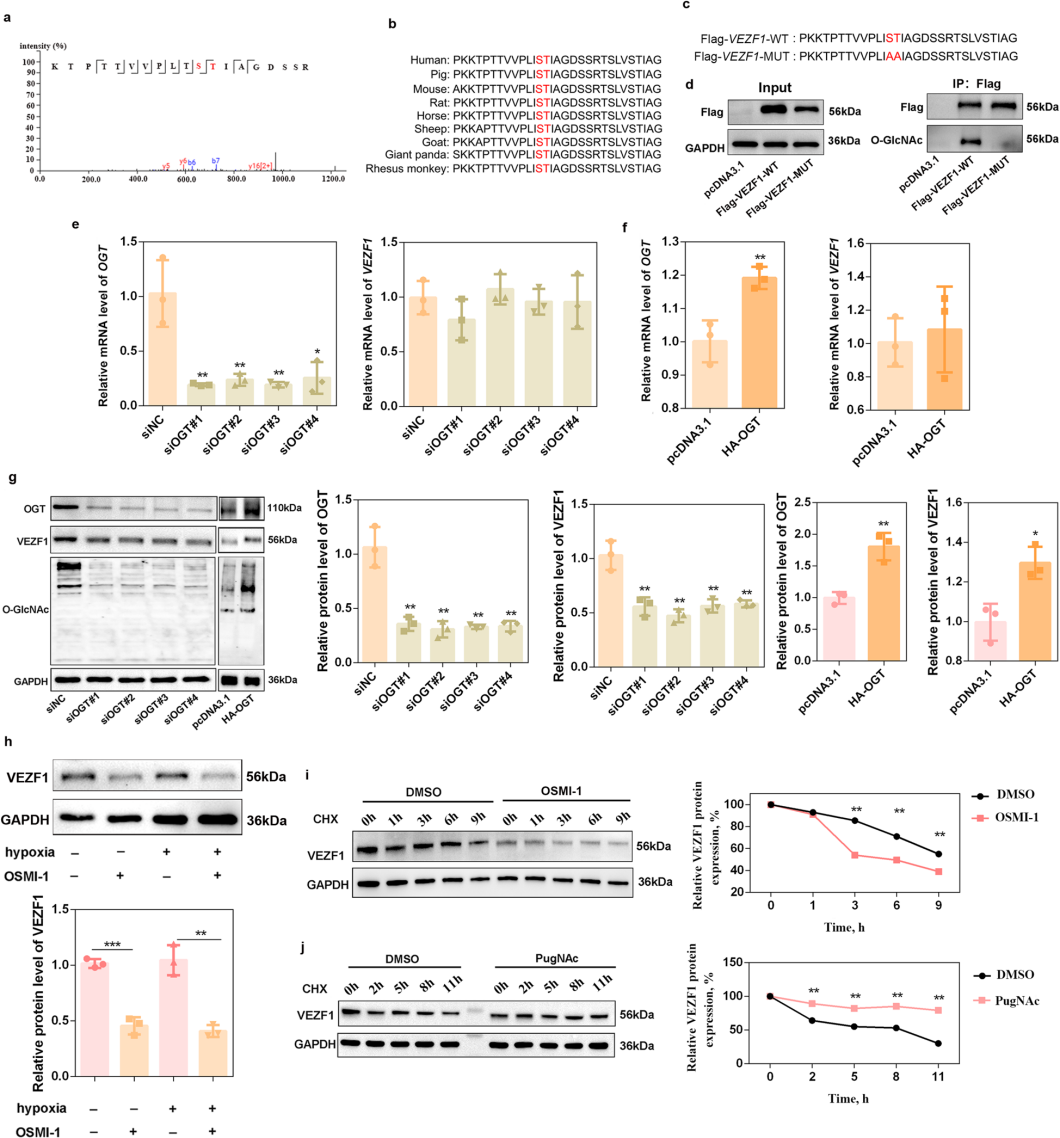
O-GlcNAcylation stabilizes the VEZF1 protein.** a** Identification of amino acid residues involved in VEZF1 O-GlcNAcylation. **b** Protein sequence alignment of VEZF1 in different species. **c** Sequences of the Flag-*VEZF1*-WT or Flag-*VEZF1*-MUT constructs. **d** Co-IP analysis was performed to detect the O-GlcNAcylation levels of the WT-*VEZF1* and MUT-*VEZF1* constructs.* n* = 3 biological replicates per group. **e** The mRNA levels of *OGT* and *VEZF1* were detected after *OGT* siRNA transfection.* n* = 3 biological replicates per group. **f** The mRNA levels of *OGT* and *VEZF1* were detected after OGT overexpression plasmid transfection. *n* = 3 biological replicates per group. **g** Western blot analysis was performed to detect OGT expression, VEZF1 expression and O-GlcNAcylation levels after OGT knockdown or overexpression.* n* = 3 biological replicates per group.** h** Western blot analysis was performed to detect VEZF1 expression after porcine GCs were treated with OSMI-1 under normoxic or hypoxic conditions. *n* = 3 biological replicates per group. **i** Analysis of the VEZF1 protein degradation rate after OSMI-1 treatment. Porcine GCs were treated with OSMI-1 and then with CHX, and the protein level of VEZF1 was recorded at 0, 1, 3, 6 and 9 h by western blot. **j** Analysis of the VEZF1 protein degradation rate after PugNAc treatment. Porcine GCs were treated with PugNAc and then with CHX, and the protein level of VEZF1 was recorded at 0, 2, 5, 8 and 11 h by western blotting. ^*^P < 0.05, ^**^P < 0.01, ^***^P < 0.001

VEZF1 is a transcription factor previously shown to regulate vasculogenesis and angiogenesis in endothelial cells [23]; our results showed that VEZF1 could be O-GlcNAcylated at the S117 and T118 sites (Fig. 4a and Table S6). Sequence alignment analysis revealed that both S117 and T118 were conserved across multiple species (Fig. 4b). To further confirm the O-GlcNAcylated sites of VEZF1, the Flag-*VEZF1*-WT sequence or the Flag-*VEZF1*-MUT sequence (Fig. 4c) were constructed and transfected into porcine GCs. As shown in Fig. 4d, the transfection of *VEZF1*-MUT plasmids caused a decrease in Flag protein and VEZF1 O-GlcNAcylation. OGT knockdown significantly decreased *OGT* mRNA expression (*P* < 0.05) without affecting *VEZF1* mRNA expression (*P* > 0.05) (Fig. 4e), and OGT overexpression significantly increased *OGT* mRNA expression (*P* < 0.01) without affecting *VEZF1* mRNA expression (*P* > 0.05) (Fig. 4f). However, the protein level of VEZF1 significantly decreased (*P* < 0.01) along with decreased O-GlcNAcylation after OGT siRNA transfection and significantly increased (*P* < 0.05) along with increased O-GlcNAcylation after OGT overexpression plasmid transfection (Fig. 4g). OSMI-1, an OGT inhibitor, was also used to decrease O-GlcNAcylation. Our results revealed that the protein expression of VEZF1 was significantly downregulated after OSMI-1 treatment (*P* < 0.01) (Fig. 4h). In addition, the addition of OSMI-1 promoted the proteolytic degradation of VEZF1 (*P* < 0.01) (Fig. 4i), whereas PugNAc, an OGA inhibitor, inhibited the proteolytic degradation of VEZF1 (*P* < 0.01) (Fig. 4j), indicating that the O-GlcNAcylation of VEZF1 is critical for its protein stability.

### HIF1A knockdown promotes porcine granulosa cell apoptosis by regulating O-GlcNAcylation-mediated VEZF1 expression

**Fig. 5:**
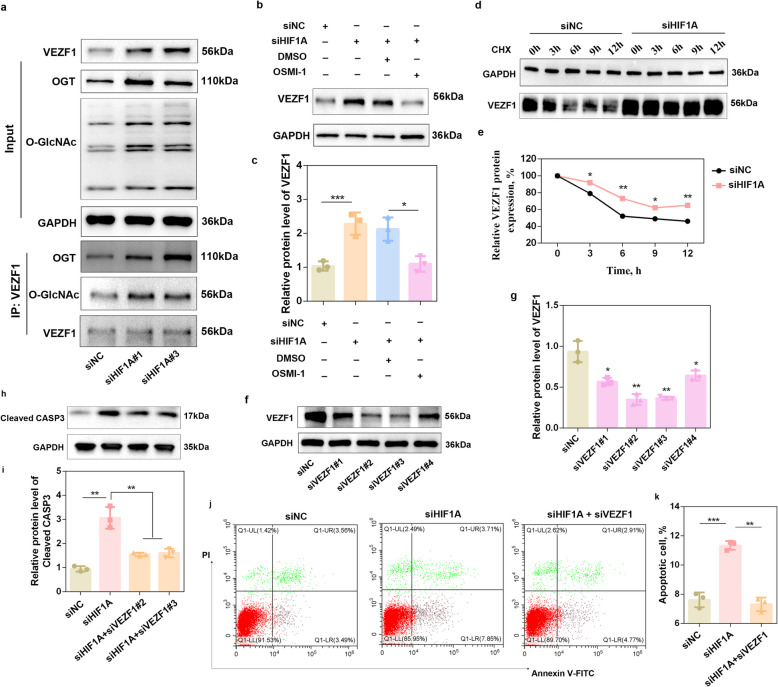
HIF1A knockdown promotes porcine granulosa cell apoptosis by regulating O-GlcNAcylation-mediated VEZF1 expression.** a** O-GlcNAcylation of VEZF1 was determined after porcine GCs were transfected with siHIF1A#1 and siHIF1A#3 under hypoxic conditions. *n* = 3 biological replicates per group. **b** and **c** Western blot analysis was performed to detect VEZF1 expression after porcine GCs were cotreated with siHIF1A and OSMI-1 under hypoxic conditions. *n* = 3 biological replicates per group. **d** and **e** Analysis of the VEZF1 protein degradation rate after siHIF1A transfection. Porcine GCs transfected with siHIF1A were treated with CHX, and the protein level of VEZF1 was recorded at 0, 3, 6, 9 and 12 h by western blotting. **f** and **g** The knockdown efficiency of the VEZF1 protein was determined after transfection with VEZF1 siRNAs.* n* = 3 biological replicates per group. **h** and **i** Western blot analysis was performed to detect cleaved CAPS3 expression after porcine GCs were cotreated with siHIF1A and siVEZF1 under hypoxic conditions. *n* = 3 biological replicates per group. **j** and **k** The apoptotic rate of porcine GCs was determined after porcine GCs were cotreated with siHIF1A and siVEZF1 under hypoxic conditions. *n* = 3 biological replicates per group. ^*^P < 0.05, ^**^P < 0.01, ^***^P < 0.001

As shown in Fig. 3, transfection of siHIF1A caused a marked increase in OGT and O-GlcNAcylation; therefore, we speculated that knockdown of HIF1A might regulate VEZF1 expression via O-GlcNAcylation. Consistent with our expectations, compared with the siNC group, the siHIF1A#1 and siHIF1A#3 groups presented significantly increased VEZF1 protein levels (*P* < 0.01) accompanied by significant increases in OGT and O-GlcNAc levels (*P* < 0.05) (Fig. 5a and Fig. S4a–c). Co-IP analysis revealed that knockdown of HIF1A increased the interaction of VEZF1 with OGT or O-GlcNAc (Fig. 5a and Fig. S4d and e). To further determine whether HIF1A affects the expression of VEZF1 through O-GlcNAcylation, we co-treated porcine GCs with siHIF1A and OSMI-1. As shown in Fig. 5b and c, OSMI-1 treatment inhibited the upregulation of VEZF1 induced by siHIF1A transfection (*P* < 0.05). In addition, VEZF1 degradation was suppressed in response to siHIF1A transfection after CHX treatment compared with that in the siNC group (Fig. 5d and e).

We next explored whether VEZF1 mediates the pro-apoptotic role of siHIF1A. The results revealed that siVEZF1#2 and siVEZF1#3 most effectively knocked down the protein expression of VEZF1 (*P* < 0.01) (Fig. 5f–g). The results of siHIF1A and siVEZF1 co-transfection showed that, compared with siHIF1A, knocking down VEZF1 decreased cleaved CASP3 expression (Fig. 5h–i) and rescued the apoptotic rate of GCs (Fig. 5j–k) (*P* < 0.01).

### The HIF1A-VEZF1 signaling axis modulates porcine granulosa cell apoptosis through ET-1-mediated inhibition of FOXO1

**Fig. 6:**
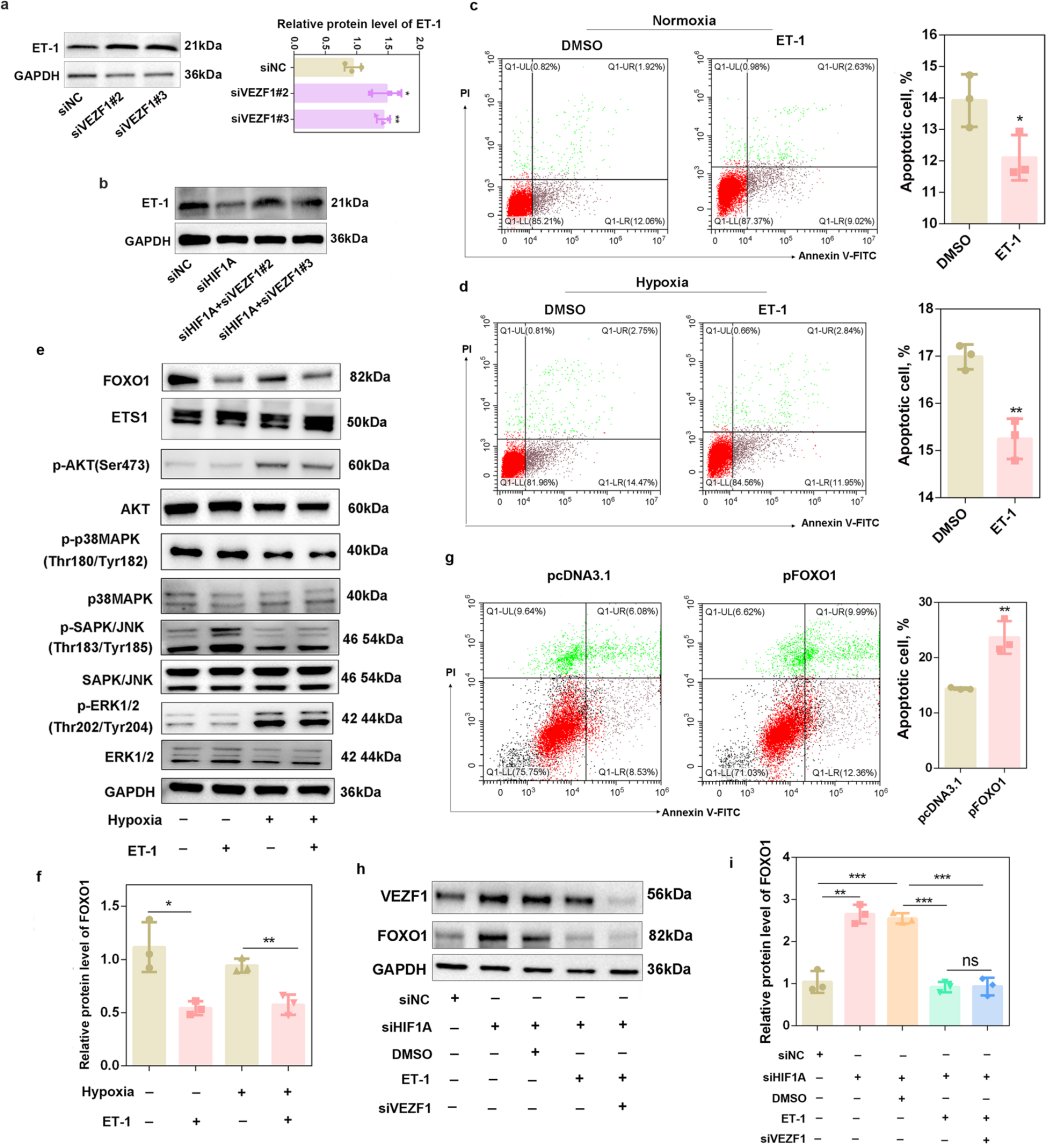
The HIF1A-VEZF1 signaling axis modulates porcine granulosa cell apoptosis through ET-1-mediated inhibition of FOXO1.** a** Western blot analysis was performed to detect ET-1 expression after porcine GCs were transfected with siVEZF1#2 and siVEZF1#3. *n* = 3 biological replicates per group. **b** Western blot analysis was performed to detect ET-1 expression after porcine GCs were cotransfected with siHIF1A and siVEZF1. *n* = 3 biological replicates per group. **c** The apoptotic rate of porcine GCs was determined after ET-1 treatment under normoxic conditions.* n* = 3 biological replicates per group. **d** The percentage of apoptotic porcine GCs was determined after ET-1 treatment under hypoxic conditions. *n* = 3 biological replicates per group. **e** and **f** Western blot analysis was performed to detect the FOXO1, EST, AKT, p38MAPK, JNK, and ERK signaling pathways. **g** The percentage of apoptotic porcine GCs was determined after FOXO1 overexpression.* n* = 3 biological replicates per group. **h** and **i** Western blot analysis was performed to detect VEZF1 and FOXO1 protein levels in porcine GCs cotreated with siHIF1A, ET-1 and siVEZF1. ^*^P < 0.05, ^**^P < 0.01, ^***^P < 0.001, ns, not significant

To investigate the molecular mechanism by which the HIF1A-VEZF1 axis modulates the apoptosis of porcine GCs, we explored the downstream effectors of VEZF1. ET-1 is a strongly vasoconstrictive protein that is encoded by endothelin-1 (*EDN1*) [24, 25]. Previous studies have demonstrated that VEZF1 transcriptionally regulates *EDN1* promoter activity by binding to the “ACCCCCA” motif, thereby controlling ET-1 expression in human endothelial cells [26, 27]. Intriguingly, the expression of ET-1 was found to be negatively correlated with follicular atresia in porcine ovaries [28]. To explore whether VEZF1 regulates the expression of *EDN1* as a transcription factor in porcine GCs, we explored the promoter sequences of pig *EDN1* and identified the “ACCCCCA” motif (Fig. S5a). The results of the dual-luciferase assay for pGL3-*EDN1*-WT and pGL3-*EDN1*-MUT (Fig. S5b) revealed that VEZF1 did not increase the fluorescence activity of the *EDN1* promoter (*P* > 0.05) compared with that of the pcDNA3.1 group, and the mutation did not reduce the fluorescence activity of the *EDN1* promoter (*P* > 0.05) compared with that of the WT group (Fig. S5c), indicating that VEZF1 cannot regulate the activity of the *EDN1* promoter. Unexpectedly, knockdown of VEZF1 significantly increased the mRNA expression of *EDN1* (*P* < 0.05) (Fig. S5d) and markedly increased the ET-1 protein level (*P* < 0.05) (Fig. 6a). Moreover, knockdown of HIF1A decreased the ET-1 protein level (*P* < 0.05), and siVEZF1 rescued the protein level of ET-1 (*P* < 0.05) induced by siHIF1A transfection (Fig. 6b and Fig. S5e). The addition of ET-1 significantly decreased the proportion of apoptotic porcine GCs under both normoxic (*P* < 0.05) (Fig. 6c) and hypoxic conditions (*P* < 0.01) (Fig. 6d).

ET-1 functions as an autocrine/paracrine growth factor [29], coordinating the simultaneous activation of multiple downstream signaling pathways (e.g., MAPK/ERK/JNK, PI3K/Akt, E26 transformation-specific 1 (ETS1), and FOXO1) [30–32]. To explore the downstream factors of ET-1 that regulate its anti-apoptotic effect in porcine GCs, we tested the protein levels of FOXO1, ETS1, p-AKT/AKT, p-p38MAPK/p38MAPK, p-JNK/JNK, and p-ERK/ERK after ET-1 treatment via western blot analysis. The results revealed that the activity of the AKT, ERK, p38MAPK and ETS1 pathways was not regulated by ET-1, whereas the activity of JNK was upregulated by ET-1 only under normoxic conditions (Fig. 6e). In contrast, ET-1 significantly decreased FOXO1 expression under both normoxic (*P* < 0.05) and hypoxic (*P* < 0.01) conditions (Fig. 6e–f).

FOXO1 is a crucial mediator of the stress response in ovarian GCs, and its role in porcine GC apoptosis has been well documented [33, 34]. Consistent with previous studies, our results showed that FOXO1 overexpression (Fig. S5f) significantly increased the proportion of apoptotic porcine GCs (Fig. 6g). As a downstream component of the HIF1A-VEZF1 axis, FOXO1 was regulated by HIF1A or VEZF1 siRNAs (*P* < 0.01) (Fig. S5g–h). Furthermore, the significant upregulation of FOXO1 induced by HIF1A siRNA (*P* < 0.01) could be abolished by ET-1 treatment (*P* < 0.001), and siVEZF1 transfection did not further exacerbate the decrease in FOXO1 after ET-1 treatment (Fig. 6h and i); these results suggest that ET-1 is the main target of the HIF1A-VEZF1 axis in regulating FOXO1 expression.

### FOXO1 transactivates *BAX* expression to promote porcine granulosa apoptosis

**Fig. 7:**
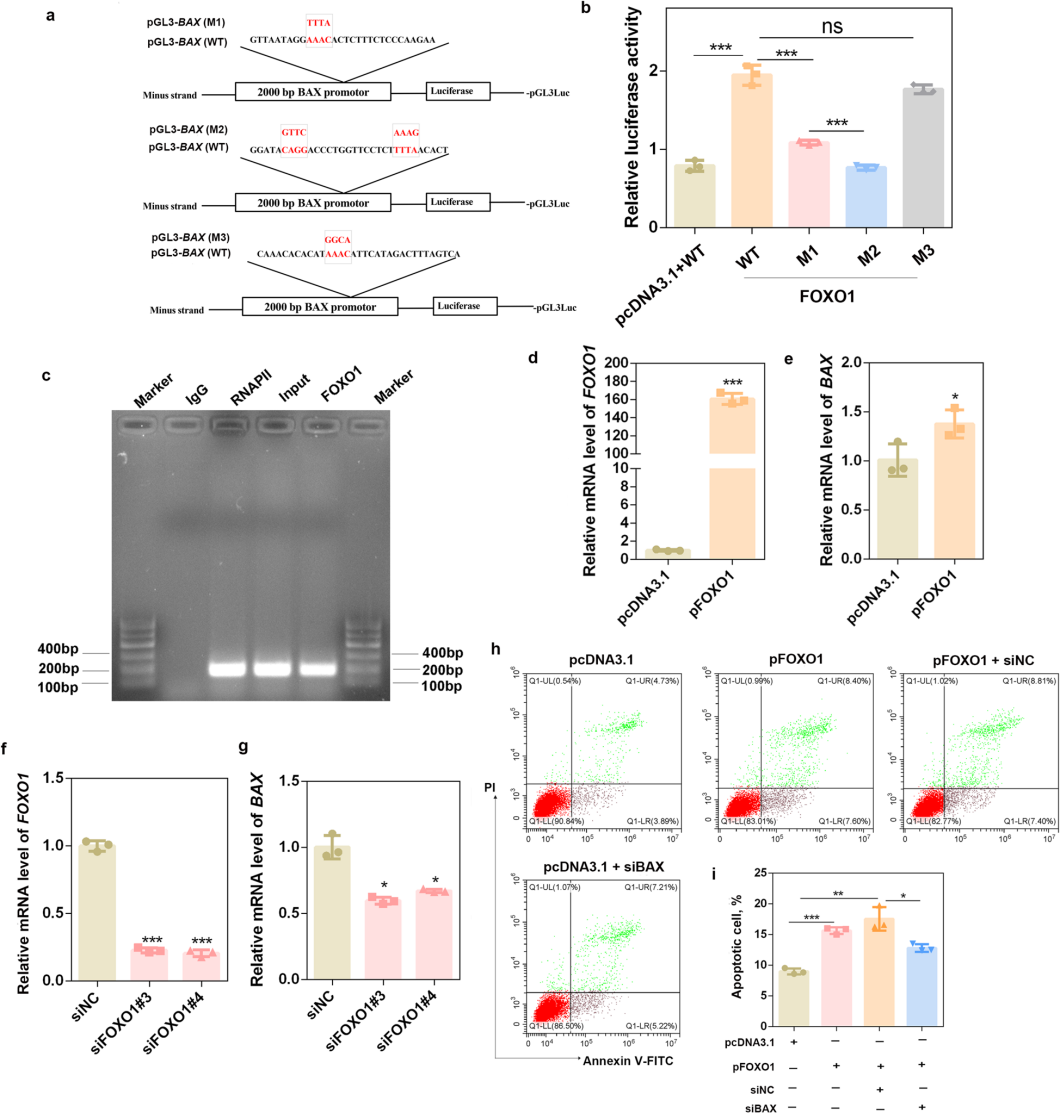
FOXO1 transactivates *BAX* expression to promote porcine granulosa apoptosis. **a** Sequences of the *BAX* promoter for the pGL3-*BAX*-WT, pGL3-*BAX*-M1, pGL3-*BAX*-M2 and pGL3-*BAX-*M3 constructs. **b** A dual-luciferase assay was performed to detect the binding of FOXO1 to the *BAX* promoter. *n* = 3 biological replicates per group. **c** Binding of FOXO1 to the *BAX* promoter in porcine GCs was detected with ChIP assays. **d** and **e** The mRNA levels of *FOXO1* and *BAX* were detected after *FOXO1* overexpression. *n* = 3 biological replicates per group. **f** and **g** The mRNA levels of *FOXO1* and *BAX* were detected after FOXO1 siRNA transfection in porcine GCs.*n* = 3 biological replicates per group. **h** and **i** The apoptotic rate was determined after porcine GCs were cotransfected with the *FOXO1* overexpression plasmid and *BAX* siRNA. *n* = 3 biological replicates per group. ^*^P < 0.05, ^**^P < 0.01, ^***^P < 0.001, ns, not significant

FOXO1 is a well-known apoptosis-inducing factor that transcriptionally activates apoptosis-related genes such as *Noxa* and *Fasl* in mice [35, 36]. In porcine GCs, FOXO1 regulates cell cycle progression, promotes apoptosis, and acts as an autophagy inducer [10]. However, the downstream genes directly associated with FOXO1-mediated apoptosis in porcine GCs remain unclear. We analyzed the expression of apoptosis-related genes (*FASL*, *TRAIL*, *PMAIP1*, and *BAX*) across porcine tissues and found that only *PMAIP1* and *BAX* were expressed in porcine ovaries (Fig. S6a). Sequence analysis revealed three potential FOXO1-binding sites in the *BAX* promoter (Fig. S6b). To determine whether FOXO1 directly activates the *BAX* promoter, we co-transfected GCs with pGL3-*BAX* WT or MUT plasmids (Fig. 7a). The results of the dual-luciferase reporter assay revealed that, compared with the control, FOXO1 overexpression significantly increased *BAX* promoter activity (*P* < 0.001) (Fig. 7b). Mutation of FRE1 (M1) and FRE2 (M2) attenuated (*P* < 0.001) *BAX* promoter activity, whereas mutation of FRE3 (M3) had no significant effect (*P* > 0.05) compared with the WT group (Fig. 7b), indicating that FOXO1 binds to FRE1 and FRE2 to activate *BAX* promoter activity in porcine GCs. ChIP assays further confirmed the binding of FOXO1 to the *BAX* promoter region; the negative control (IgG) resulted in no nonspecific amplification, whereas the positive control, input, and FOXO1 antibody groups all successfully amplified the *BAX* promoter (Fig. 7c). Consistently, FOXO1 overexpression upregulated *BAX* mRNA levels (*P* < 0.05) (Fig. 7d and e), whereas FOXO1 silencing via siRNA transfection (Fig. S6c and d) significantly reduced *BAX* expression (*P* < 0.05) (Fig. 7f and g).

BAX, a pro-apoptotic protein, plays a crucial role in promoting programmed cell death by permeabilizing the mitochondrial outer membrane and releasing pro-apoptotic factors into the cytosol. Our results revealed that overexpression of *BAX* (Fig. S7a) increased GC apoptosis (*P* < 0.01) (Fig. S7b and c). More importantly, co-treatment with *BAX* siRNAs (siBAX) and FOXO1 overexpression plasmids (Fig. S7d and e) decreased GC apoptosis compared with the co-treatment group of siNC and FOXO1 overexpression plasmids (*P* < 0.05) (Fig. 7h and i).

### The HIF1A-VEZF1-ET-1 signaling axis modulates *BAX* expression by regulating FOXO1 nuclear expression

**Fig. 8:**
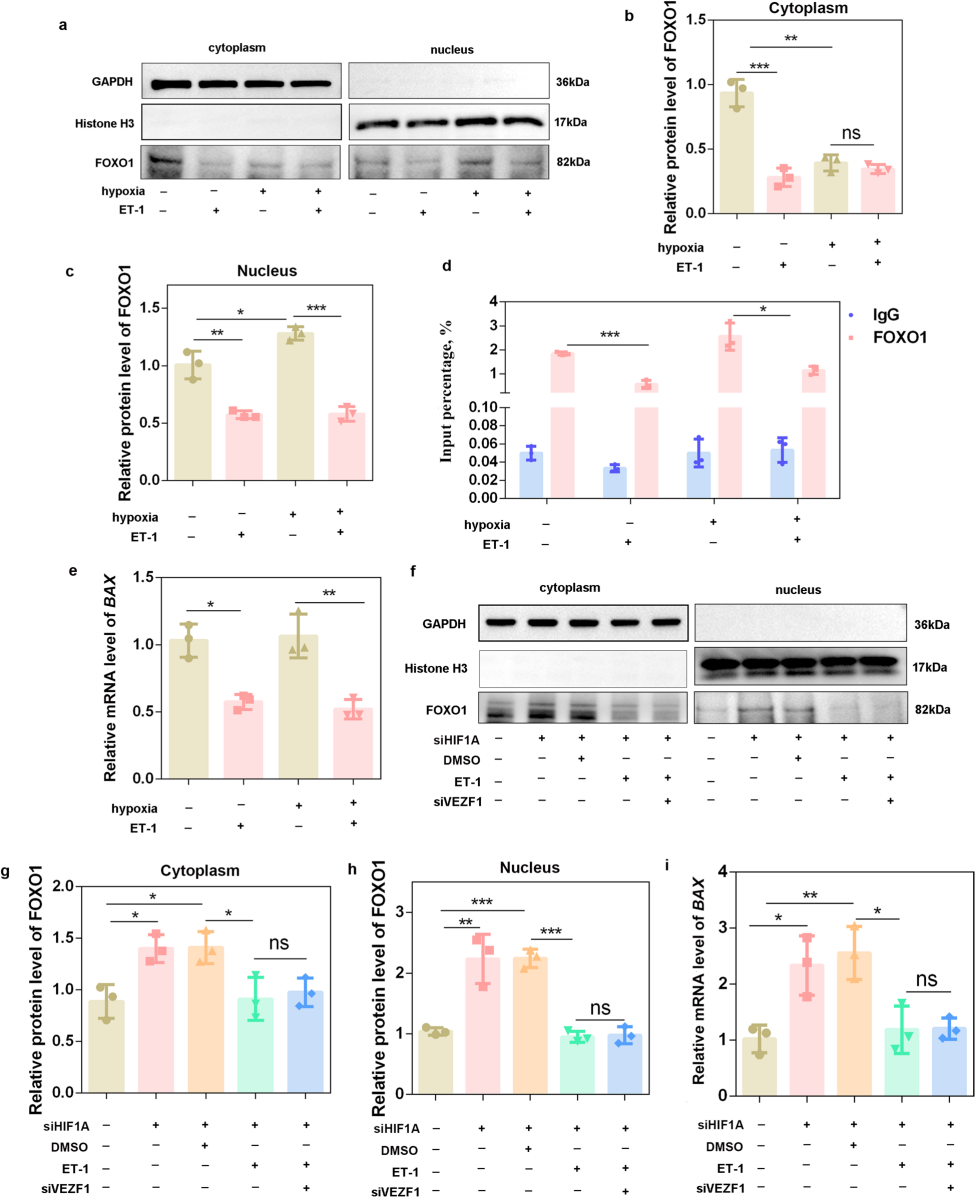
The HIF1A-VEZF1-ET-1 signaling axis modulates *BAX* expression by regulating FOXO1 nuclear expression. **a‒c** Nuclear‒cytoplasmic fractionation was performed to analyze FOXO1 expression in the cytoplasm and nucleus after porcine GCs were treated with ET-1 for 24 h. *n* = 3 biological replicates per group. **d** ChIP‒qPCR assays were performed to detect the binding of FOXO1 to the *BAX* promoter in porcine GCs following ET-1 treatment under normoxic or hypoxic conditions. *n* = 3 biological replicates per group. **e** The mRNA levels of *BAX* were detected following ET-1 treatment under normoxic or hypoxic conditions. *n* = 3 biological replicates per group. **f‒h** Nuclear‒cytoplasmic fractionation was performed to analyze FOXO1 expression in the cytoplasm and nucleus after porcine GCs were cotreated with siHIF1A, ET-1 and siVEZF1. *n* = 3 biological replicates per group. **i** The mRNA levels of *BAX* were detected after porcine GCs were cotreated with siHIF1A, ET-1 and siVEZF1. *n* = 3 biological replicates per group. ^*^P < 0.05, ^**^P < 0.01, ^***^P < 0.001, ns, not significant

Transcription factors must be localized in the nucleus to exert transcriptional regulation [37]. Nuclear‒cytoplasmic fractionation results showed that hypoxic treatment decreased cytoplasmic FOXO1 protein levels (*P* < 0.01) and increased nuclear FOXO1 protein levels (*P* < 0.05) (Fig. 8a–c). Compared with the control, ET-1 treatment significantly reduced nuclear FOXO1 expression under both normoxic (*P* < 0.01) and hypoxic (*P* < 0.001) conditions (Fig. 8a and c). ChIP‒qPCR further confirmed that ET-1 markedly decreased FOXO1 binding to the *BAX* promoter (Fig. 8d), accompanied by a significant reduction in *BAX* mRNA expression (Fig. 8e) under both conditions (*P* < 0.05).

Next, we speculated that HIF1A, the upstream regulator that modulates VEZF1-ET-1-FOXO1 signaling, regulates BAX expression by modulating FOXO1 nuclear expression. Our results revealed that the knockdown of HIF1A significantly increased the BAX mRNA and protein levels (Fig. S8). Knockdown of HIF1A increased the nuclear and cytoplasmic FOXO1 abundance, and ET-1 reduced the nuclear FOXO1 expression induced by siHIF1A transfection (*P* < 0.001). Transfection of siVEZF1 did not further reduce ET-1-mediated FOXO1 nuclear inhibition (*P* > 0.05) (Fig. 8f–h), which is consistent with the total FOXO1 protein levels (Fig. 6i). In addition, siHIF1A transfection upregulated *BAX* mRNA expression; however, this effect was completely reversed by ET-1 treatment (*P* < 0.05), and siVEZF1 did not potentiate ET-1-mediated *BAX* repression (*P* > 0.05) (Fig. 8i).

## Discussion

HIF1A is activated and primarily influences cellular proliferation and function by regulating adaptive responses to hypoxic conditions in multiple cell types. Feng et al*.* [38] demonstrated that HIF1A recruited coactivator-associated arginine methyltransferase 1, which occupies the promoters of genes critically involved in the cell cycle and the Wnt signaling pathway, to modulate the proliferation and invasion of triple-negative breast cancer cells. In the bovine ovary, *HIF1A* mRNA levels are markedly elevated during the terminal maturation phase of follicular development [39]. In this study, we observed high *HIF1A* expression in porcine ovarian tissues. Furthermore, HIF1A is predominantly expressed in GCs and oocytes, which is consistent with the oxygen gradient within ovarian follicles. GCs express FSHR to mediate FSH signaling, and HIF1A facilitates FSH-dependent ovulation and oocyte health by activating mitophagy [17]. Although reduced HIF1A expression is associated with PCOS [40], direct evidence linking HIF1A to follicular selection remains limited. Here, the downregulation of *HIF1A* in atretic follicles, coupled with its positive correlation with estrogen synthesis markers (FSHR, ESR1), underscores its protective role against follicular atresia. These findings align with prior studies highlighting the role of HIF1A-mediated angiogenesis and glycolysis in supporting GC growth under hypoxia.

O-GlcNAcylation is involved in cellular stress responses. Under hypoxic conditions, O-GlcNAcylation mediates the activation of HIF1A [41], suggesting that HIF1A acts as a downstream effector of O-GlcNAcylation. Moreover, the regulatory relationships among hypoxia, HIF1A, and Ο-GlcNAcylation are complex and bidirectional. In C2C12 myotubes, hypoxia induces HIF1A-mediated transcriptional activation of the *OGT* promoter through hypoxia response elements (HREs), thereby increasing *OGT* mRNA levels [42]. Additionally, in bovine retinal vascular endothelial cells, a time-dependent increase in global O-GlcNAcylation levels was observed under hypoxic conditions (1%–3% O_2_) [43]. In contrast to previous studies, our findings demonstrated that hypoxia induced site-specific alterations—rather than a global increase—in the O-GlcNAcylation profiles of porcine GCs. Intriguingly, siRNA-mediated *HIF1A* knockdown increased OGT protein expression, resulting in abnormal O-GlcNAcylation accumulation; these results are consistent with the findings of Liu et al. [22], who reported opposite expression patterns between HIF1A and OGT under hypoxic conditions. As an important Functional protein in regulating protein modification, the degradation mechanism of OGT has been widely explored; researchers have reported that all BTRC, histone lysine demethylase 2, cell division cycle 20, and F-box only protein 31 regulate OGT, acting as E3 ubiquitin ligases [22, 44–46]; among them, BTRC specifically mediates the degradation of OGT under hypoxic conditions. We found that HIF1A knockdown significantly decreased the combination of BTRC with OGT to promote OGT expression under hypoxia, which indicates that HIF1A is required to maintain hypoxia-responsive O-GlcNAc homeostasis by regulating OGT degradation. Notably, our study mapped 87 O-GlcNAc-modified peptides in porcine GCs, which play critical roles in GC growth and cellular homeostasis. This comprehensive profiling provides a molecular basis for elucidating the regulatory axis between O-GlcNAc dynamics and follicular atresia.

VEZF1 is a transcription factor implicated in angiogenesis [23]. Das et al. [47] reported that it is a binding partner of Ets variant 2, and their interactions regulated the expression of hematoendothelial genes during mesodermal differentiation. Shi et al. [48] reported that VEZF1 transcriptionally activated progestin and adipoQ receptor 4 to accelerate hepatocellular carcinoma progression. To date, studies on the function of VEZF1 have focused mainly on cancer cells or endothelial cells, and its role in follicular development is unclear. Additionally, the posttranslational modifications of the VEZF1 protein remain poorly understood. In this study, O-GlcNAcymic data revealed that VEZF1 could be O-GlcNAcylated and that the O-GlcNAcylation of VEZF1 inhibited its protein degradation. HIF1A knockdown upregulates VEZF1 expression by affecting VEZF1 O-GlcNAcylation. More importantly, the knockdown of VEZF1 rescued the apoptosis of granulosa cells caused by HIF1A deficiency. Our functional assays revealed a previously unrecognized link between HIF1A deficiency and exacerbated GC apoptosis via the O-GlcNAcylation-VEZF1 axis; this is the first report of VEZF1 regulation by O-GlcNAcylation, and we identified posttranslational modifications of the VEZF1 protein, which is important for VEZF1 functional studies in the future.

The anti-apoptotic function of ET-1 in porcine follicles has been reported [27]. Flores et al*.* [28] revealed that most atretic follicles showed no detectable ET-1 expression, whereas ET-1-positive follicles were predominantly nonatretic in porcine follicles; this may be due to the role of ET-1 in preventing progesterone production in antral follicle GCs. Interestingly, as a type of endothelin, ET-2 was also found to be regulated by HIF1A [49]. In this study, we found that ET-1 mediated the HIF1A-VEZF1 axis to protect porcine GCs from apoptosis; these observations highlight the potential role of endothelin in promoting follicular survival and maintaining ovarian function during follicular selection. However, the regulatory mechanism between VEZF1 and ET-1 still needs to be further investigated. Treatment with ET-1 suppressed the expression of FOXO1, an ovarian follicle atresia- and GC apoptosis-related transcription factor. Researchers have reported that FOXO1 induces cell cycle arrest and apoptosis in porcine GCs by mediating the JNK pathway or inducing the transcription of downstream genes [10]. Although FOXO1 induces *Noxa* and *Fasl* expression in mouse GCs [35, 36], whether it directly regulates apoptosis-related genes in porcine GCs remains unclear. In this study, we found that FOXO1 directly transactivates the *BAX* promoter to promote *BAX* expression and that the HIF1A-VEZF1-ET-1 axis regulates porcine GC apoptosis via FOXO1-mediated *BAX* expression.

## Conclusion

Taken together, these findings confirm the critical role of HIF1A in follicular selection and provide a mechanistic basis for hypoxia-regulated follicular selection by connecting HIF1A and O-GlcNAcylation, which will advance our understanding of ovarian physiology and may inspire innovative strategies to improve female reproductive health and livestock breeding.

## Supplementary Information

Additional file 1: Table S1 Antibody information. Table S2 ELISA standard curves for E2 and P4. Table S3 Primer sequences for qRT‒PCR. Table S4 Primer sequences for plasmid construction. Table S5 Antibody correspondence for Co-IP analysis. Table S6 Summary of O-GlcNAcylation sequencing data.

Additional file 2: Fig. S1. Fluorescence intensity analysis of TUNEL and HIF1A in HF and AF group. Fig. S2 Hypoxic treatment promotes HIF1A expression and induces porcine GC apoptosis. Fig. S3 Dynamic changes of O-GlcNAcylation after hypoxic treatment. Fig. S4 Knockdown of HIF1A promotes VEZF1 O-GlcNAcylation. Fig. S5 The HIF1A-VEZF1 signaling axis modulates FOXO1 expression via ET-1. Fig. S6 FOXO1 is a potential regulator of *BAX* in porcine GCs. Fig. S7 Overexpression of BAX promotes porcine GC apoptosis. Fig. S8 Knockdown of HIF1A promotes BAX expression.

## Data Availability

O-GlcNAcylation 4D-label-free quantitative proteomic sequencing data have been submitted to iProX with ProteomeXchange ID: PXD064769.

## Abbreviations

AF: Atretic follicles
AR: Androgen receptor
BAX: Bcl-2 associated X
BTRC: Beta-transducin repeat containing protein
ChIP: Chromatin immunoprecipitation
CHX: Cycloheximide
CYP11A1: Cytochrome P450 family 11 subfamily A member 1
DAPI: 4',6-Diamidino-2-phenylindole-dihydro-chloride
E2: 17β-Estradiol
EDN1: Endothelin-1
ELISA: Enzyme-linked immunosorbent assay
ESR1: Estrogen receptor 1
ET-1: Endothelin-1
FASL: Fas ligand
FF: Follicular fluid
FOXO1: Forkhead box O1
FREs: FOXO1-recognized elements
FSHR: Follicle stimulating hormone receptor
GC: Granulosa cell
HDLBP: High-density lipoprotein binding protein
HF: Healthy follicle
HIF1A: Hypoxia‐inducible factor 1 subunit alpha
IB: Immunoblotting
IF: Immunofluorescence
IP: Immunoprecipitation
MUT: Mutation
OGA: O-GlcNAcase
O-GlcNAc: O-linked N-acetylglucosamine
O-GlcNAcylation: O-linked N-acetylglucosamine modification
OGT: O-linked N-acetylglucosamine (GlcNAc) transferase
P4: Progesterone
Pan-Kla: Lysine lactylation of proteins
PCOS: Polycystic ovary syndrome
PI: Propidium iodide
PMAIP1: Phorbol-12-myristate-13-acetate-induced protein 1
POF: Premature ovarian failure
SD: Standard deviation
S: Serine
sWGA: Succinylated wheat germ agglutinin
T: Threonine
TRAIL: TNF superfamily member 10
VEGF1: Vascular endothelial growth factor 1
VEZF1: Vascular endothelial zinc finger 1
WCL: Whole-cell lysates
WT: Wild type
ZKSCAN1: Zinc finger protein with KRAB, SCAN domains 1
