# Supplementary material for: HIF1A regulates follicular atresia through O-GlcNAcylation-mediated VEZF1/ET-1/FOXO1/BAX signaling in porcine granulosa cells
Source: J Anim Sci Biotechnol. 2025 Sep 20;16:127. doi: 10.1186/s40104-025-01263-0 (PMC12449798; doi:10.1186/s40104-025-01263-0)
Supplement: Supplementary file 1 — Additional file 1: Table S1 Antibody information. Table S2 ELISA standard curves for E2 and P4. Table S3 Primer sequences for qRT‒PCR. Table S4 Primer sequences for plasmid construction. Table S5 Antibody correspondence for Co-IP analysis. Table S6 Summary of O-GlcNAcylation sequencing data. [file 40104_2025_1263_MOESM1_ESM.docx]

**Table S1 Antibody information**

| **Full Protein Name** | **Abbreviation** | **Company** | **Cat. No.** | **Dilution** |
| --- | --- | --- | --- | --- |
| Hypoxia inducible factor 1 subunit alpha | HIF1A | CST | 36169 | 1:500 for WB |
| Hypoxia inducible factor 1 subunit alpha | HIF1A | CST | 36169 | 1:50 for IF |
| Glyceraldehyde-3-phosphate dehydrogenase | GAPDH | Proteintech | 60004-1-Ig | 1:2000 for WB |
| Caspase 3 | CASP3 | Proteintech | 25128-1-AP | 1:1000 for WB |
| O- linked N-acetylglucosamine | O-GlcNAc | CST | 9875 | 1:1000 for WB |
| lysine lactylation of proteins | Pan-Kla | PTM BIO | PTM-1401 | 1:1000 for WB |
| Histone H3 | Histone H3 | CST | 9715 | 1:2000 for WB |
| O-GlcNAc transferase | OGT | Proteintech | 11576-2-AP | 1:1000 for WB |
| beta-transducin repeat containing E3 ubiquitin protein ligase | BTRC | Proteintech | 28393-1-AP | 1:2000 for WB |
| beta-transducin repeat containing E3 ubiquitin protein ligase | BTRC | Proteintech | 28393-1-AP | 1:100 for CO-IP |
| vascular endothelial zinc finger 1 | VEZF1 | Santa Cruz | sc-365560 | 1:1000 for WB |
| vascular endothelial zinc finger 1 | VEZF1 | Santa Cruz | sc-365560 | 1:20 for CO-IP |
| high density lipoprotein binding protein | HDLBP | Proteintech | 15406-1-AP | 1:2000 for WB |
| zinc finger with KRAB and SCAN domains 1 | ZKSCAN1 | Proteintech | 25214-1-AP | 1:2000 for WB |
| endothelin 1 | ET-1 | Proteintech | 12191-1-AP | 1:1000 for WB |
| DYKDDDDK tag | Flag | Proteintech | 20543-1-AP | 1:5000 for WB |
| DYKDDDDK tag | Flag | Proteintech | 20543-1-AP | 1:100 for CO-IP |
| forkhead box O1 | FOXO1 | CST | 2880 | 1:2000 for WB |
| forkhead box O1 | FOXO1 | CST | 2880 | 1:50 for ChIP |
| ETS proto-oncogene 1 | ETS1 | Proteintech | 12118-1-AP | 1:1000 for WB |
| AKT serine/threonine kinase | AKT | Proteintech | 10176-2-AP | 1:1000 for WB |
| Phospho-AKT(Ser473) | p-AKT | Proteintech | 66444-1-Ig | 1:1000 for WB |
| P38 mitogen-activated protein kinase | P38MAPK | CST | 9212 | 1:800 for WB |
| Phospho-p38 MAPK (Thr180/Tyr182) | p-p38MAPK | CST | 4511 | 1:800 for WB |
| c-Jun N-terminal Kinase | JNK | CST | 9252 | 1:1000 for WB |
| Phospho-SAPK/JNK (Thr183/Tyr185) | p-JNK | CST | 4668 | 1:1000 for WB |
| Extracellular Signal-Regulated Kinase | ERK | Proteintech | 11257-1-AP | 1:1000 for WB |
| Phospho-ERK1/2 (Thr202/Tyr204) | p-ERK | Proteintech | 28733-1-AP | 1:1000 for WB |
| Goat Anti-Rabbit IgG, HRP Conjugated | | CWBIO | CW0103S | 1：5000 for WB |
| Goat Anti-Mouse IgG, HRP Conjugated | | CWBIO | CW0102S | 1：5000 for WB |
| Goat Anti-Rabbit IgG (Alexa Fluor® 594) | | Abcam | ab150080 | 1：200  for IF |

**Table S2 ELISA standard curves for E2 and P4**

| E2 concentration (x) | 80 | 40 | 20 | 10 | 5 | 0 |
| --- | --- | --- | --- | --- | --- | --- |
| OD value  (y) | 0.1163 | 0.2094 | 0.4517 | 0.6405 | 1.1873 | 2.5374 |
| 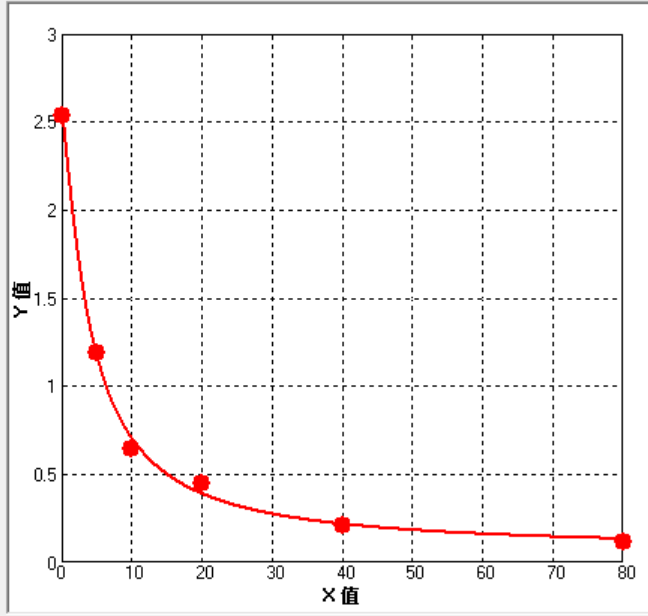y = (2.53859-0.06588) / [1+ (x/4.16609)^1.21157] + 0.06588 | | | | | | |
| R^2^ = 0.99790 | | | | | | |

| P4 concentration (x) | 20 | 10 | 5 | 2.5 | 1.25 | 0 |
| --- | --- | --- | --- | --- | --- | --- |
| OD value  (y) | 0.1269 | 0.2147 | 0.4113 | 0.6438 | 1.1932 | 2.1581 |
| 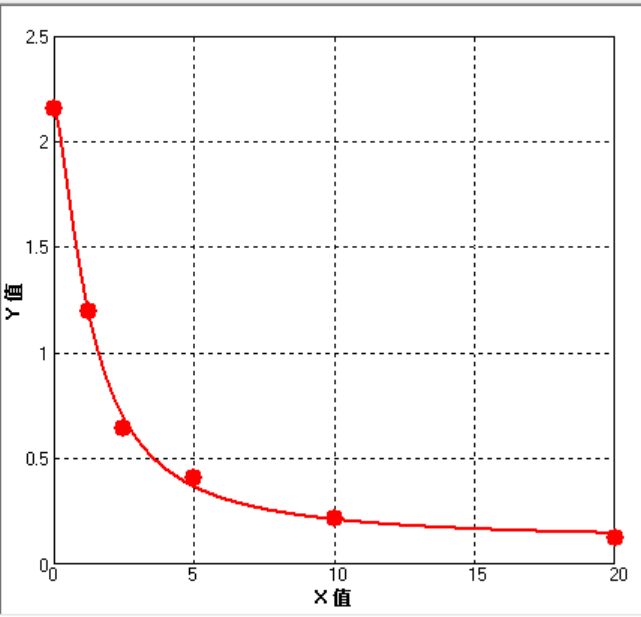y = (2.15987-0.10921) / [1+ (x/1.32130)^1.46133] + 0.10921  R^2^ = 0.99838 | | | | | | |

|  |
| --- |

**Table S3 Primer sequences for qRT-PCR**

| **Full name** | **Abbreviation** | **Gene ID** | **Primer sequence (5’-3’)** |
| --- | --- | --- | --- |
| Hypoxia inducible factor 1 subunit alpha | *HIF1A* | 396696 | F: CCTGATTGAGTGCAGGGTCA  R: TTTTGGCAGCAATGACACAGA |
| Glyceraldehyde-3-phosphate dehydrogenase | *GAPDH* | 396823 | F: GTCGGAGTGAACGGATTTGGC  R: GAACTTGCCGTGGGTGGAAT |
| BCL2 associated X | *BAX* | 396633 | F: GGCCCTTTTGCTTCAGGGTTTC  R: ACACTCGCTCAACTTCTTGGT |
| Cytochrome P450 family 11 subfamily A member 1 | *CYP11A1* | 403329 | F: GCACTGGAGAGGGAGCAT  R: TTGCCGAGCTTCTCCCTGTAA |
| Follicle stimulating hormone receptor | *FSHR* | 397679 | F: CGCGGTTGAACTGAGGTTTG  R: TGGGCAGATTGGAGAACACA |
| Estrogen receptor 1 | *ESR1* | 397435 | F: GCAAAGAGGGTGCCAGGATT  R: CTCCACCATTCCCTCGACAC |
| Androgen receptor | *AR* | 397582 | F: AGAAGTACCTGTGTGCCAGC  R: TTCAGTTTCCGAGCTCCCAG |
| Endothelin 1 | *EDN1* | 396915 | F: GGGTCAACACTCCAGAACACA  R: TGGCACACTGGCATCTATCC |
| Vascular endothelial zinc finger 1 | *VEZF1* | 100511406 | F: AGCGCTTCAAGAGGAAGGAC  R: GCATGAGGGAGGAAAATTGGC |
| Forkhead box O1 | *FOXO1* | 397077 | F: CGCTTGGACTGTGACATGGA  R: GTACTTTTCAGTGTAGCCTGCTC |
| Fas ligand | *FASL* | 396726 | F: TGGGGATGTTTCAGCTCTTCC  R: AGGGTAGATTGGGGTGACCTAT |
| TNF superfamily member 10 | *TRAIL* | 406191 | F: TCCCACCGATGACGAGAGAA  R: GAATGCCTTGTTGCTTTTCTGA |
| Phorbol-12-myristate-13-acetate-induced protein 1 | *PMAIP1* | 397278 | F: CAAAGTTCCGGCTGCTGTTT  R: ACTCGACTTCAGGATCTGGC |

**Table S4 Primer sequences for plasmid construction**

| **Primer name** |  | **sequence (5’-3’)** |
| --- | --- | --- |
| pGL3-*BAX-*WT  pGL3*-BAX*-M1  pGL3-*BAX*-M2  pGL3-*BAX*-M3 | F:  R:  F:  R:  F:  R:  F:  R: | cgagctcttacgcgtgctagcAGCCATTGTCTGACTGCAGACA  cagtaccggaatgccaagcttGGCGCGCGTCCTGTCACG  GGtttaACTCTTTCTCCCAAGAACATGCTTTCT  GGGAGAAAGAGTtaaaCCTATTAACTTTGAAGCTGGCTTTAT  tcaccctggttcctctaaagACACTGCTTTGCTTTCAGCTTATC  ttagaggaaccagggtgaacTATCCTGGAAAACTATGAGACTGTAGATG  CACAAACACACATggcaATTCATAGACTTTAGTCATCTGGAACG  TtgccATGTGTGTTTGTGTGAAATCCAAGGATC |

**Table S5 Antibody correspondence for Co-IP analysis**

| **IP antibodies** | **IB antibodies** |
| --- | --- |
| BTRC | OGT |
| sWGA | VEZF1, HDLBP, ZKSCAN1 |
| Flag | O-GlcNAc |
| VEZF1 | OGT, O-GlcNAc |

**Table S6 Summary of O-GlcNAcylation sequencing data**

| **Protein** | **Protein Name** | **Location** | **Identified or quantified** |
| --- | --- | --- | --- |
| A0A8D1SBJ8 | Zinc finger with KRAB and SCAN domains 1 | S472 | Quantified |
| A0A8D1FHM8 | Nucleoporin Nup153 | T1090 | Quantified |
| A0A8D1DE09 | Nsp1_C domain-containing protein | T13 | Quantified |
| A0A8D1DE09 | Nsp1_C domain-containing protein | T17 | Quantified |
| A0A5G2RDB9 | Forkhead box K1 | T596 | Quantified |
| A0A5G2RDB9 | Forkhead box K1 | T601 | Quantified |
| A0A8D1ZK59 | MAX gene-associated protein | S1897 | Quantified |
| A0A8D1ZK59 | MAX gene-associated protein | S1900 | Quantified |
| A0A4X1SKF9 | Vascular endothelial zinc finger 1 | T118 | Quantified |
| A0A8D1WY95 | HECT domain-containing protein | T966 | Quantified |
| A0A8D0ICK7 | ADF-H domain-containing protein | T212 | Quantified |
| A0A8D1NDK0 | Annexin | S12 | Quantified |
| A0A8D1NDK0 | Annexin | S18 | Quantified |
| A0A8D1NDK0 | Annexin | T19 | Quantified |
| A0A8D1NDK0 | Annexin | S22 | Quantified |
| A0A5G2QTP9 | Endoplasmic reticulum protein 44 | T154 | Quantified |
| A0A8D1Q5W3 | Elongation factor 1-alpha | T71 | Quantified |
| K9J4P3 | UDP-glucose glycoprotein glucosyltransferase 1 | S1393 | Quantified |
| A0A8D1ULC5 | Vimentin | S51 | Quantified |
| A0A8D0K8G7 | Aspartate--tRNA ligase, cytoplasmic | S10 | Quantified |
| A0A8D0K8G7 | Aspartate--tRNA ligase, cytoplasmic | T12 | Quantified |
| A0A480J284 | Host cell factor 1 isoform X2 | T579 | Quantified |
| A0A480J284 | Host cell factor 1 isoform X2 | T587 | Quantified |
| A0A480J284 | Host cell factor 1 isoform X2 | T579 | Quantified |
| A0A480J284 | Host cell factor 1 isoform X2 | T588 | Quantified |
| A0A8D1CMF4 | ALMS1 centrosome and basal body associated protein | S1960 | Quantified |
| A0A8D1CMF4 | ALMS1 centrosome and basal body associated protein | S1909 | Quantified |
| A0A480J284 | Host cell factor 1 isoform X2 | T779 | Quantified |
| A0A4X1SKF9 | Vascular endothelial zinc finger 1 | S117 | Quantified |
| A0A4X1SKF9 | Vascular endothelial zinc finger 1 | T118 | Quantified |
| A0A480J284 | Host cell factor 1 isoform X2 | T587 | Quantified |
| A0A480J284 | Host cell factor 1 isoform X2 | T588 | Quantified |
| A0A8D0I288 | Elongation factor 1-alpha | T279 | Quantified |
| A0A8D1S6C1 | Nucleosome-remodeling factor subunit BPTF | T2109 | Quantified |
| A0A5G2RLE0 | RNA 3'-terminal-phosphate cyclase (ATP) | S27 | Quantified |
| A0A8D0RH29 | Junction plakoglobin | T32 | Quantified |
| A0A8D1KV12 | YEATS domain containing 2 | T609 | Quantified |
| A0A8D1EUG4 | Notch receptor 2 | T675 | Quantified |
| A0A8D1HXH4 | Nuclear factor 1 | T372 | Identified |
| A0A480MLT8 | Nuclear pore complex protein Nup153 isoform 2 | S1133 | Identified |
| A0A8D0WI45 | Talin 2 | S1688 | Identified |
| A0A8D0WI45 | Talin 2 | T1691 | Identified |
| A0A480YPK0 | Protein PRRC2C isoform X5 | T2239 | Identified |
| Q8MIM5 | Transcription factor GATA-4 | S109 | Identified |
| F8T0U2 | Phosphatidylinositol-binding clathrin assembly protein | S353 | Identified |
| F8T0U2 | Phosphatidylinositol-binding clathrin assembly protein | T356 | Identified |
| F8T0U2 | Phosphatidylinositol-binding clathrin assembly protein | S359 | Identified |
| A0A8D1M0M0 | Phosphatidylinositol binding clathrin assembly protein | S353 | Identified |
| A0A8D1M0M0 | Phosphatidylinositol binding clathrin assembly protein | S362 | Identified |
| A0A8D1M0M0 | Phosphatidylinositol binding clathrin assembly protein | T363 | Identified |
| A0A8D0R019 | Nucleoporin 214 | S461 | Identified |
| A0A8D0R019 | Nucleoporin 214 | S464 | Identified |
| F1SLB3 | RNA binding motif single stranded interacting protein 2 | S288 | Identified |
| F1SLB3 | RNA binding motif single stranded interacting protein 2 | S289 | Identified |
| Q9GL01 | Dolichyl-diphosphooligosaccharide--protein glycosyltransferase subunit 2 | S93 | Identified |
| Q9GL01 | Dolichyl-diphosphooligosaccharide--protein glycosyltransferase subunit 2 | S94 | Identified |
| A0A480YPK0 | Protein PRRC2C isoform X5 | T2239 | Identified |
| A0A480YPK0 | Protein PRRC2C isoform X5 | S2242 | Identified |
| A0A8D1CMF4 | ALMS1 centrosome and basal body associated protein | T1902 | Identified |
| A0A481CRT1 | Host cell factor 1 isoform X1 | T634 | Identified |
| A0A8D0Q1Q3 | Nuclear pore complex protein Nup214 | S1993 | Identified |
| A0A8D1M7V2 | Vascular endothelial zinc finger 1 | S117 | Identified |
| A0A8D1M7V2 | Vascular endothelial zinc finger 1 | T118 | Identified |
| A0A8D1M7V2 | Vascular endothelial zinc finger 1 | S124 | Identified |
| F1RGX4 | GLOBIN domain-containing protein | S50 | Identified |
| F1RGX4 | GLOBIN domain-containing protein | S53 | Identified |
| A0A8D2BWT9 | Integrin beta | S643 | Identified |
| A0A8D2BWT9 | Integrin beta | S643 | Identified |
| A0A8D2BWT9 | Integrin beta | S648 | Identified |
| A0A8D2BWT9 | Integrin beta | S648 | Identified |
| A0A8D2BWT9 | Integrin beta | S653 | Identified |
| A0A8D0RT61 | Junction plakoglobin | T40 | Identified |
| A0A8D1RUZ7 | High density lipoprotein binding protein | S231 | Quantified |
| A0A8D1HEH7 | High density lipoprotein binding protein | S284 | Quantified |
| A0A8D0TJT9 | Vigilin | S231 | Quantified |
| A0A8D0TJ12 | Vigilin | S284 | Quantified |
| A0A287BDJ5 | Drebrin 1 | T161 | Quantified |
| A0A8D0YJB7 | ADF-H domain-containing protein | T161 | Quantified |
| A0A8D1F0F6 | ADF-H domain-containing protein | T161 | Quantified |
| A0A480P528 | Drebrin isoform X2 | T161 | Quantified |
| A0A8D0YCR1 | ADF-H domain-containing protein | T161 | Quantified |
| A0A287A0I8 | Drebrin 1 | T161 | Quantified |
| A0A480V6V1 | Drebrin isoform a | T161 | Quantified |
| A0A4X1SEQ0 | ADF-H domain-containing protein | T161 | Quantified |
| A0A8D1ZZX2 | ADF-H domain-containing protein | T161 | Quantified |
| A0A8D1WQI6 | ADF-H domain-containing protein | T161 | Quantified |
| A0A8D1QUY1 | ADF-H domain-containing protein | T161 | Quantified |
