# Supplementary material for: HIF1A regulates follicular atresia through O-GlcNAcylation-mediated VEZF1/ET-1/FOXO1/BAX signaling in porcine granulosa cells
Source: J Anim Sci Biotechnol. 2025 Sep 20;16:127. doi: 10.1186/s40104-025-01263-0 (PMC12449798; doi:10.1186/s40104-025-01263-0)
Supplement: Supplementary file 2 — Additional file 2: Fig. S1. Fluorescence intensity analysis of TUNEL and HIF1A in HF and AF group. Fig. S2 Hypoxic treatment promotes HIF1A expression and induces porcine GC apoptosis. Fig. S3 Dynamic changes of O-GlcNAcylation after hypoxic treatment. Fig. S4 Knockdown of HIF1A promotes VEZF1 O-GlcNAcylation. Fig. S5 The HIF1A-VEZF1 signaling axis modulates FOXO1 expression via ET-1. Fig. S6 FOXO1 is a potential regulator of BAX in porcine GCs. Fig. S7 Overexpression of BAX promotes porcine GC apoptosis. Fig. S8 Knockdown of HIF1A promotes BAX expression. [file 40104_2025_1263_MOESM2_ESM.docx]

**Fig. S1**


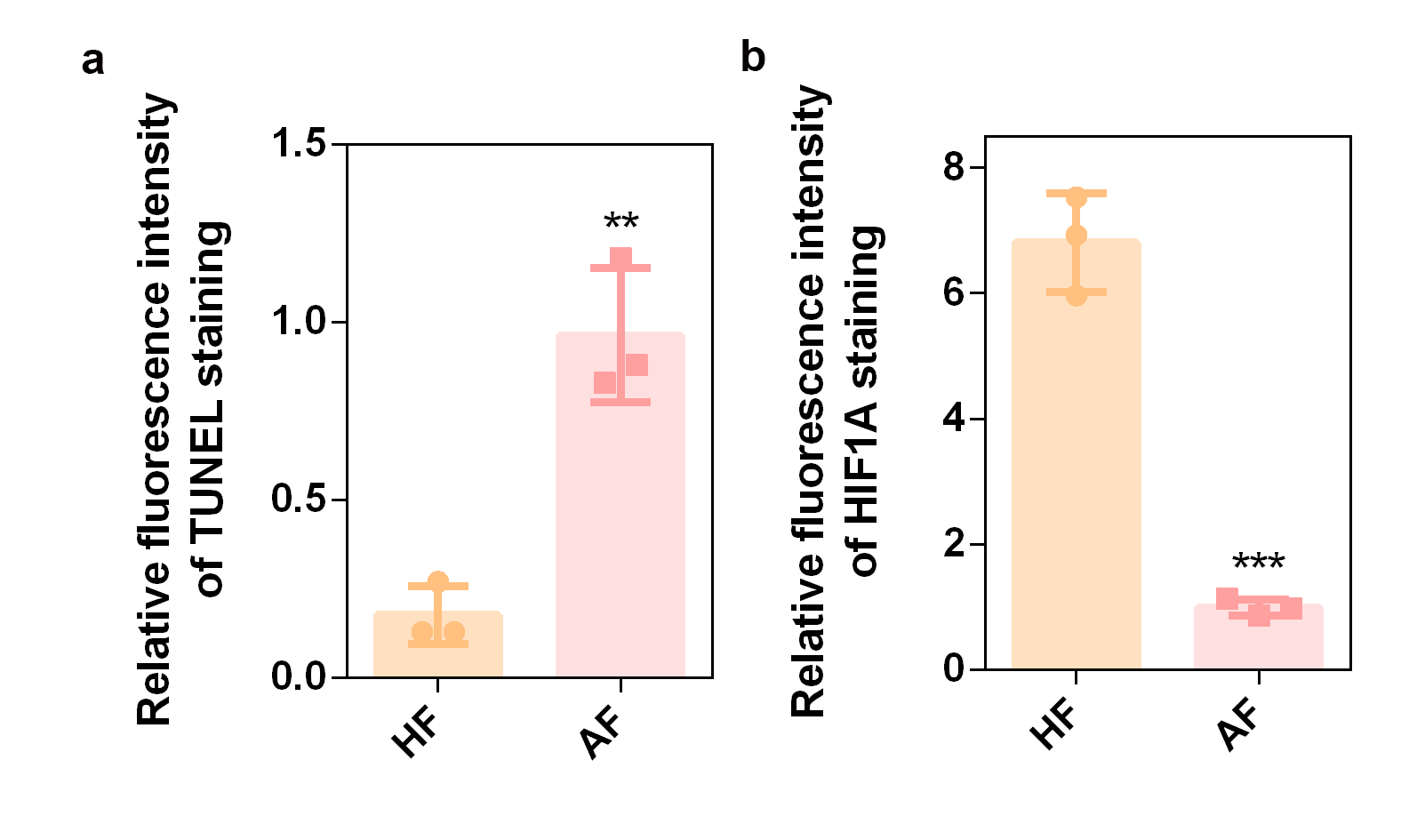


**Fig S1.** Fluorescence intensity analysis of TUNEL and HIF1A in HF and AF group. **a** Fluorescence intensity of TUNEL in HF and AF. *n* = 3 biological replicates per group. **b** Fluorescence intensity of HIF1A in HF and AF. *n* = 3 biological replicates per group. ***P* < 0.01, ****P* < 0.001.

**Fig. S2**


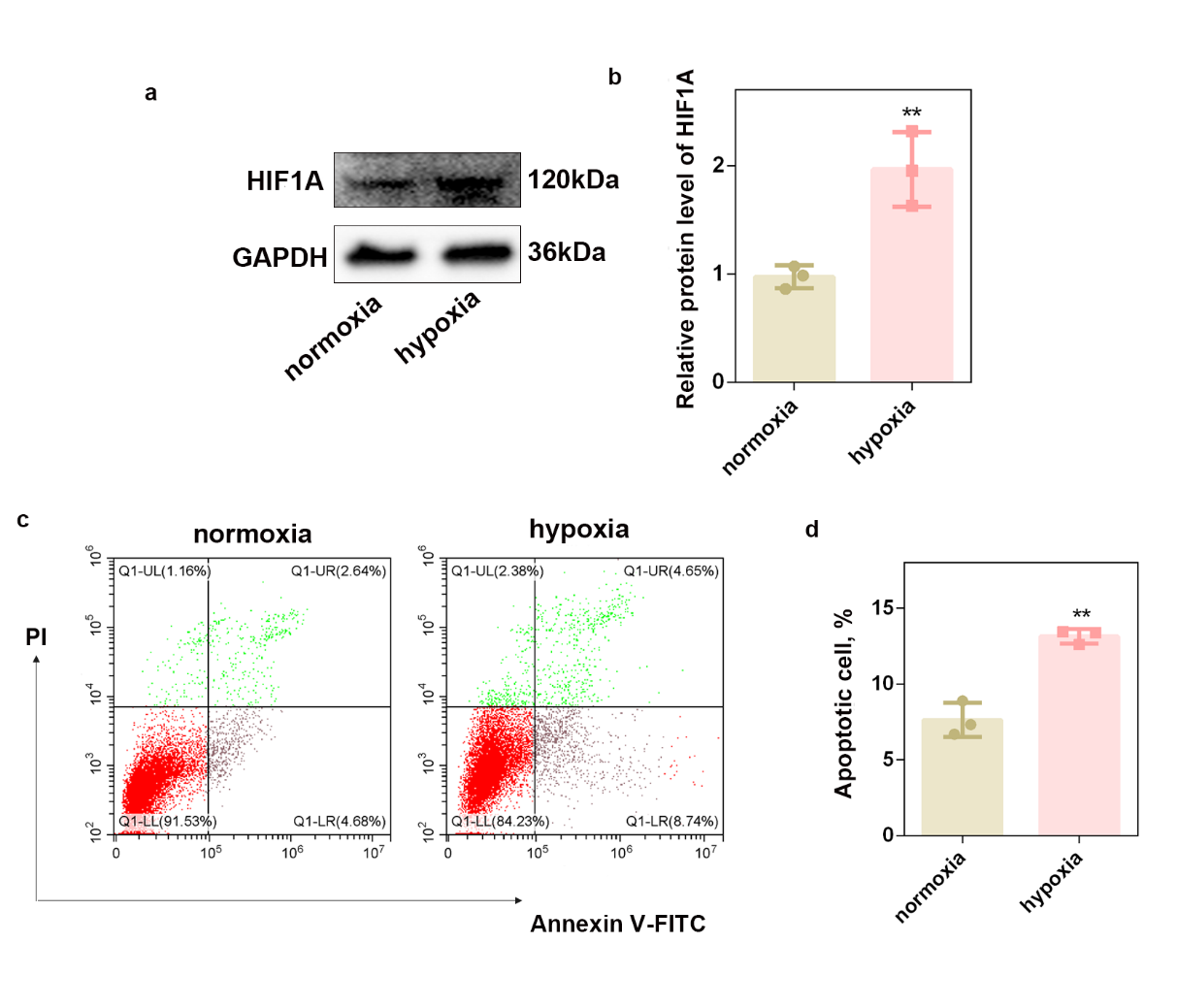


**Fig. S2** Hypoxic treatment promotes HIF1A expression and induces porcine GC apoptosis. **a, b** Western blot analysis was performed to detect HIF1A expression after hypoxic treatment. *n* = 3 biological replicates per group. **c, d** The apoptotic rate of porcine GCs was determined after hypoxic treatment. *n* = 3 biological replicates per group. ***P* < 0.01.

**Fig. S3**


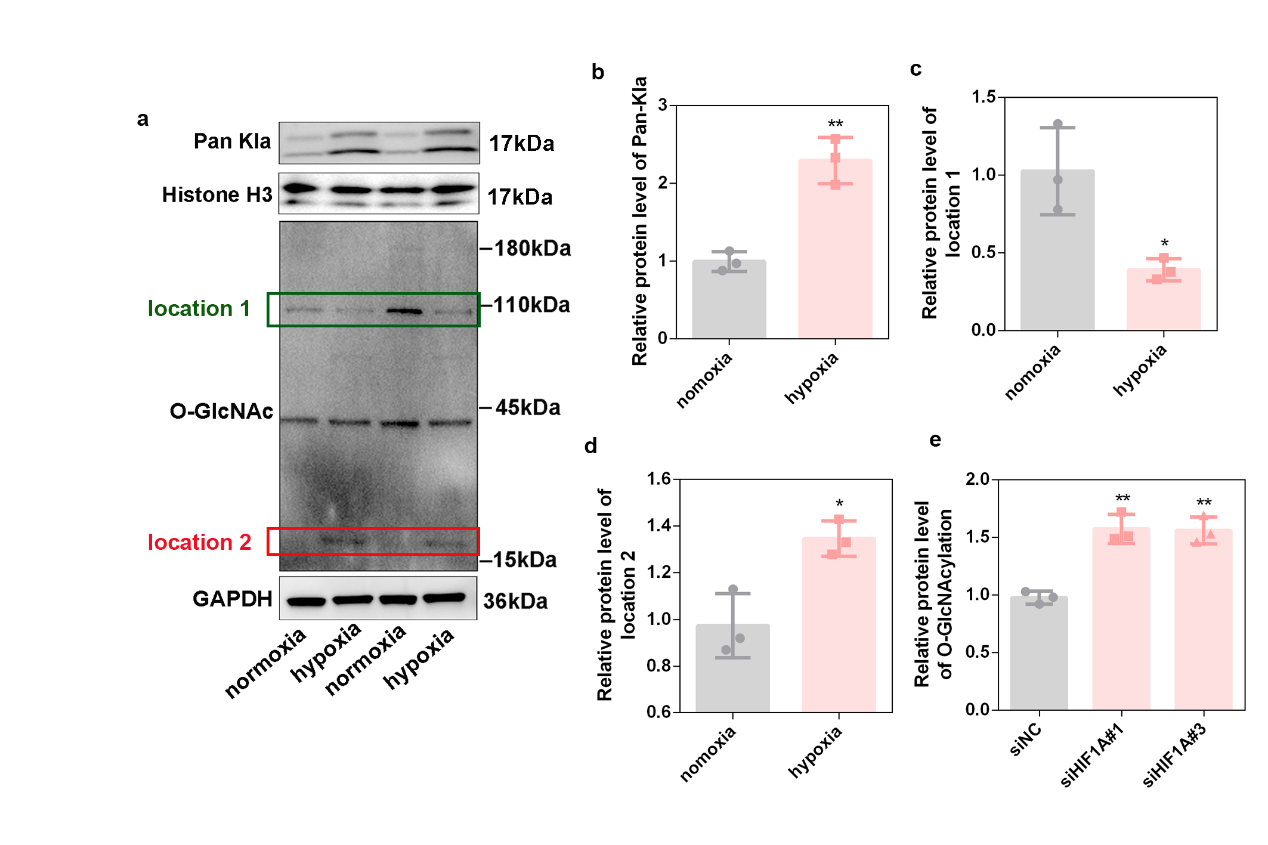


**Fig. S3** Dynamic changes of O-GlcNAcylation after hypoxic treatment. **a-d** Porcine GCs were cultured under normoxic or hypoxic conditions for 24 h, and then the protein expression of Pan-Kla and O-GlcNAc was analyzed by western blot. Histone H3 was used as the loading control for Pan-Kla, and GAPDH was used as the loading control for O-GlcNAc. *n* = 3 biological replicates per group. **e** Western blot analysis was performed to detect O-GlcNAcylation levels after HIF1A siRNAs transfection. *n* = 3 biological replicates per group. **P* < 0.05, ***P* < 0.01.

**Fig. S4**


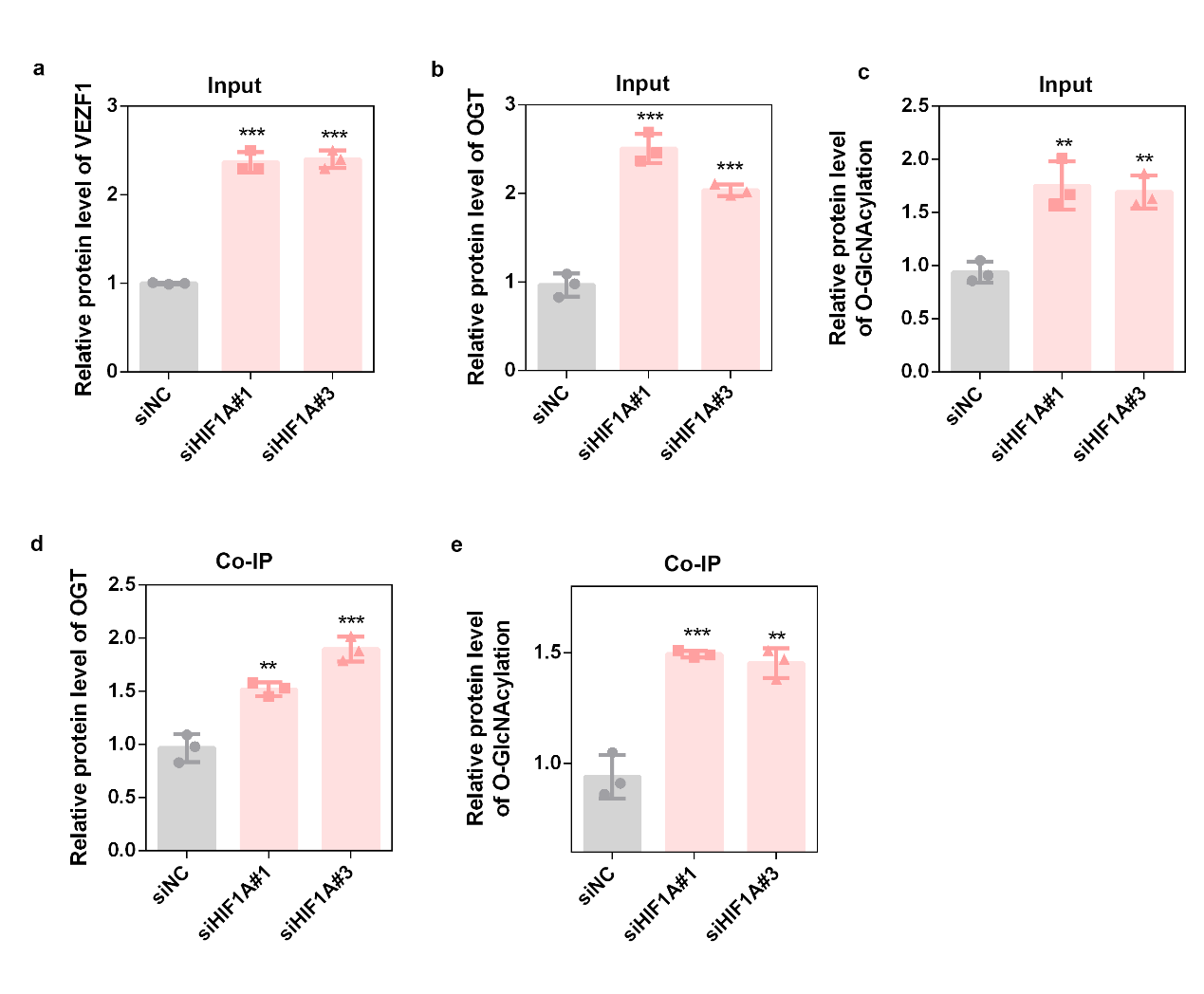


**Fig. S4** Knockdown of HIF1A promotes VEZF1 O-GlcNAcylation. **a-c** The protein levels of VEZF1, OGT and O-GlcNAcylation were analyzed after siHIF1A#1 and siHIF1A#3 transfection. *n* = 3 biological replicates per group. **d, e** Co-IP analysis was performed to detect the combination of VEZF1 with OGT or O-GlcNAc after siHIF1A#1 and siHIF1A#3 transfection. *n* = 3 biological replicates per group. ***P* < 0.01, ****P* < 0.001.

**Fig. S5**


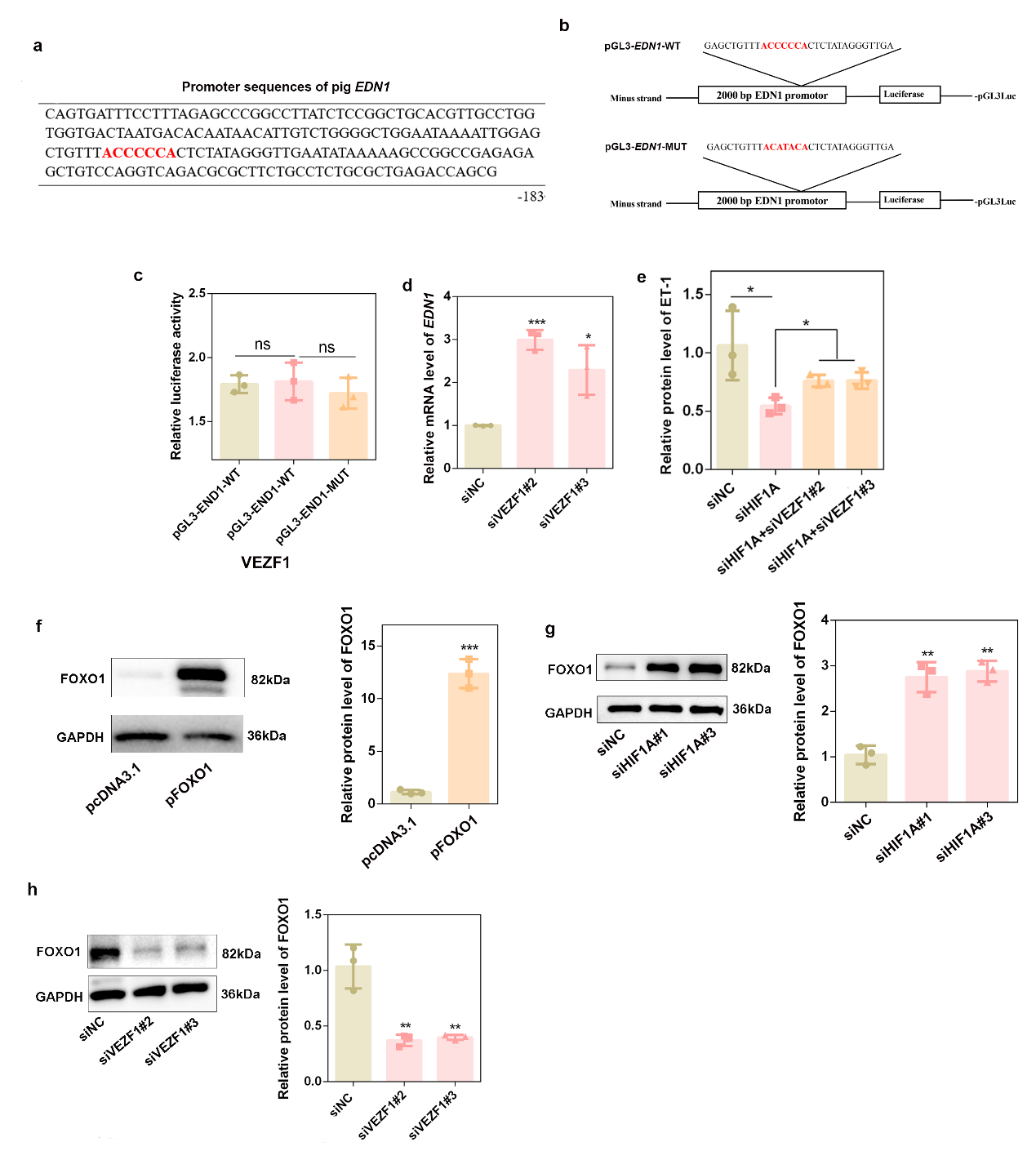


**Fig. S5** The HIF1A-VEZF1 signaling axis modulates FOXO1 expression via ET-1. **a** The sequences of porcine *EDN1* promoter. **b** The sequences of pGL3-*EDN1*-WT and pGL3-*EDN1*-MUT constructs. **c** Dual-luciferase assay was performed to detect the binding of VEZF1 to the *EDN1* promoter. *n* = 3 biological replicates per group. **d** The mRNA level of *EDN1* was detected after siVEZF1 transfection. *n* = 3 biological replicates per group. **e** The protein level of ET-1 was analyzed after siHIF1A and siVEZF1 co-transfection. *n* = 3 biological replicates per group. **f** The protein level of FOXO1 was detected after FOXO1-overexpression plasmid transfection. *n* = 3 biological replicates per group. **g** The protein levels of FOXO1 after siHIF1A#1 and siHIF1A#3 transfection. *n* = 3 biological replicates per group. **h** The protein levels of FOXO1 were analyzed after siVEZF1#2 and siVEZF1#3 transfection. *n* = 3 biological replicates per group. **P* < 0.05, ***P* < 0.01, ****P* < 0.001, ns, not significant.

**Fig. S6**


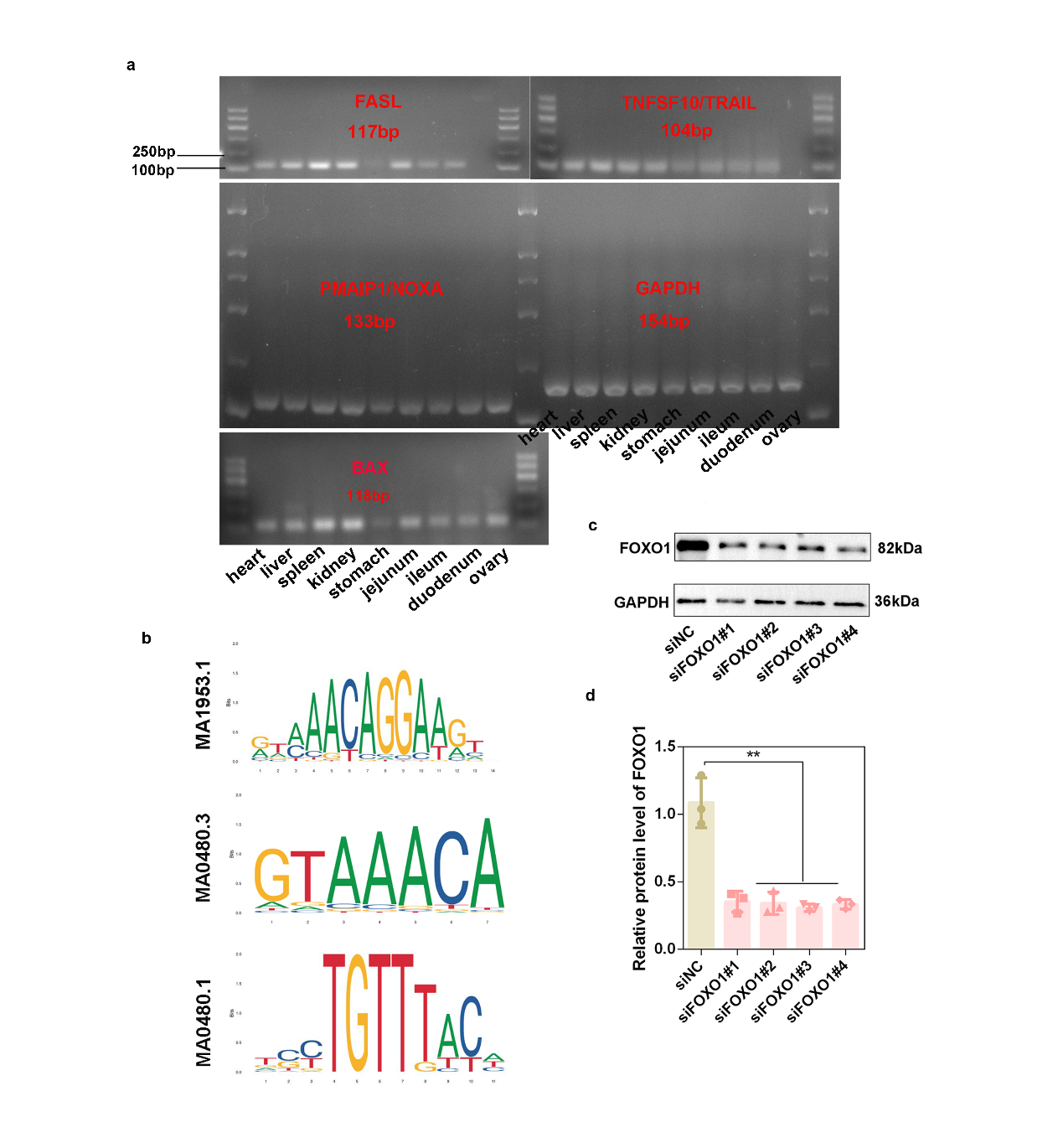


**Fig. S6** FOXO1 is a potential regulator of *BAX* in porcine GCs. **a** The expression patterns of apoptosis-related genes (*FASL*, *TRAIL*, *PMAIP1*, and *BAX*) were determined by RT-PCR across porcine tissues. **b** The predicted binding sites between FOXO1 and *BAX* promoter by JASPAR. **c, d** The protein levels of FOXO1 after siFOXO1 transfection were analyzed by western blot. GAPDH was used as the loading control. Relative FOXO1 protein levels were calculated by ImageJ following the indicated treatments. *n* = 3 biological replicates per group. ***P* < 0.01.

**Fig. S7**


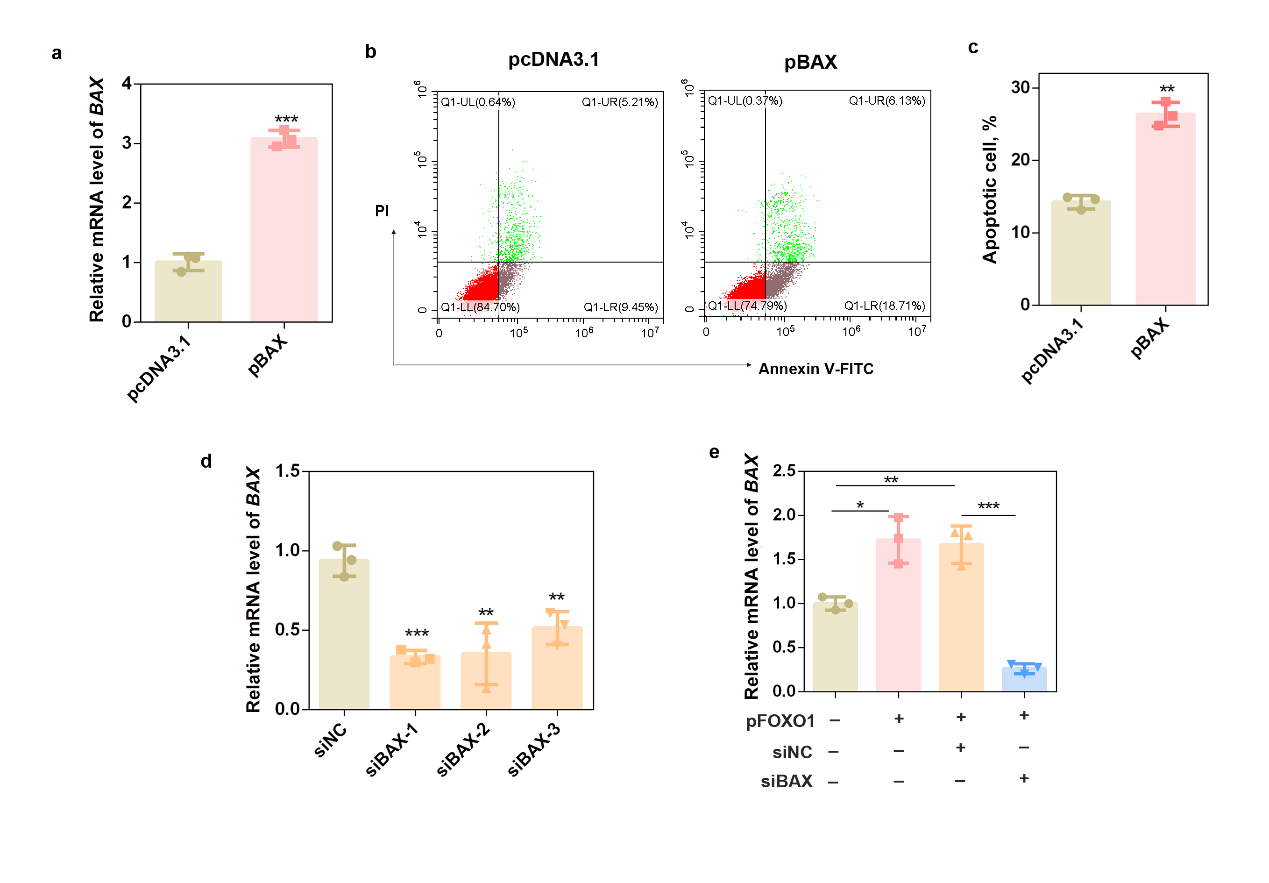


**Fig. S7** Overexpression of BAX promotes porcine GC apoptosis. **a** The overexpression efficiency of *BAX* after porcine GCs were transfected with *BAX* overexpression plasmid. *n* = 3 biological replicates per group. **b, c** The apoptotic rate of porcine GCs was determined after *BAX* overexpression. *n* = 3 biological replicates per group. **d** The knockdown efficiency of *BAX* after porcine GCs were transfected with *BAX* siRNAs. *n* = 3 biological replicates per group. **e** The mRNA expression of *BAX* was detected after porcine GCs were co-transfected with the combination of FOXO1 overexpression plasmid and siBAX. *n* = 3 biological replicates per group. **P* < 0.05, ***P* < 0.01, ****P* < 0.001.

**Fig. S8**


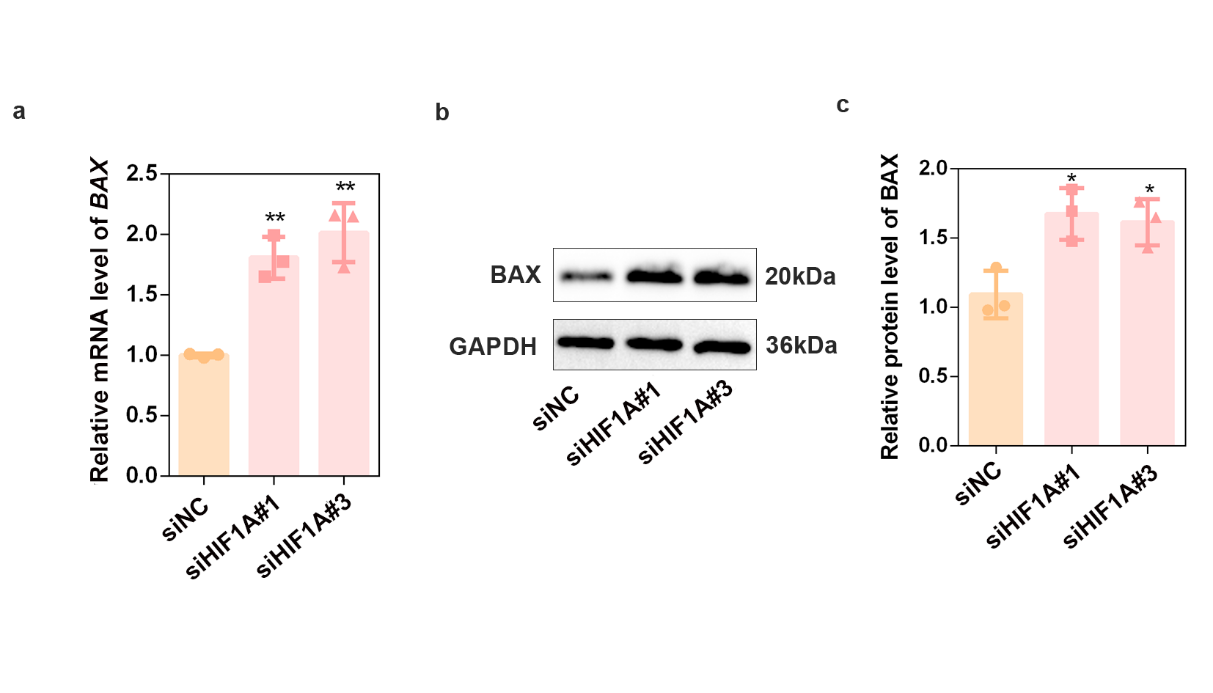


**Fig. S8** Knockdown of HIF1A promotes BAX expression. **a** The mRNA level of *BAX* was assessed after porcine GCs were transfected with siHIF1A#1 and siHIF1A#3 under hypoxia conditions. *n* = 3 biological replicates per group. **b, c** Porcine GCs were transfected with siHIF1A#1 and siHIF1A#3 under hypoxia conditions, and the protein level of BAX was detected by western blot. GAPDH was used as the loading control. Relative BAX protein levels were calculated by ImageJ following the indicated treatments. *n* = 3 biological replicates per group. **P* < 0.05, ***P* < 0.01.
